# Structural basis for the pore-forming activity of a complement-like toxin

**DOI:** 10.1126/sciadv.adt2127

**Published:** 2025-03-28

**Authors:** Bronte A. Johnstone, Michelle P. Christie, Riya Joseph, Craig J. Morton, Hamish G. Brown, Eric Hanssen, Tristan C. Sanford, Hunter L. Abrahamsen, Rodney K. Tweten, Michael W. Parker

**Affiliations:** ^1^ Department of Biochemistry and Pharmacology, Bio21 Molecular Science and Biotechnology Institute, University of Melbourne, Parkville, VIC 3010, Australia.; ^2^ ARC Centre for Cryo-Electron Microscopy of Membrane Proteins, Bio21 Molecular Science and Biotechnology Institute, University of Melbourne, Parkville, VIC 3010, Australia.; ^3^ Ian Holmes Imaging Centre, Bio21 Molecular Science & Biotechnology Institute, University of Melbourne, Parkville, VIC 3010, Australia.; ^4^ Department of Microbiology & Immunology, The University of Oklahoma Health Sciences Center, Oklahoma City, OK, USA.; ^5^ Australian Cancer Research Foundation Rational Drug Discovery Centre, St Vincent’s Institute of Medical Research, Fitzroy, VIC 3065, Australia.

## Abstract

Pore-forming proteins comprise a highly diverse group of proteins exemplified by the membrane attack complex/perforin (MACPF), cholesterol-dependent cytolysin (CDC), and gasdermin superfamilies, which all form gigantic pores (>150 angstroms). A recently found family of pore-forming toxins, called CDC-like proteins (CDCLs), are wide-spread in gut microbes and are a prevalent means of antibacterial antagonism. However, the structural aspects of how CDCLs assemble a pore remain a mystery. Here, we report the crystal structure of a proteolytically activated CDCL and cryo–electron microscopy structures of a prepore-like intermediate and a transmembrane pore providing detailed snapshots across the entire pore-forming pathway. These studies reveal a sophisticated array of regulatory features to ensure productive pore formation, and, thus, CDCLs straddle the MACPF, CDC, and gasdermin lineages of the giant pore superfamilies.

## Teaser

A detailed mechanism by which a pore-forming toxin forms gigantic membrane pores using structural biology is reported.

## INTRODUCTION

Pore-forming proteins (PFPs) play key roles in living organisms with many involving the secretion of water-soluble protomers to form a transmembrane pore on a target cell. During this process, the components undergo complex conformational changes and intermediary steps. In the β class of PFPs, the pores range from 20 to 30 Å in the case of the aerolysin family (*1*–*3*) to larger pores that range from 80 to 120 Å for the membrane attack complex (MAC)/perforin (MACPF) family (*4*–*10*) through 150 to 215 Å for gasdermin (GSDM) pores (*11*–*15*) to greater than 250 Å in the cholesterol-dependent cytolysin (CDC) family (*16*–*20*) and some bacterial GSDMs (bGSDMs) (*11*). Members of the MACPF and CDC families (*21*, *22*) share a common topology consisting of a core, twisted β sheet associated with two α-helical bundles (α-HBs) that form transmembrane hairpins (TMHs) in the pore state. For the MACPFs, numerous structures of the pore state have been obtained (*4*, *6*–*10*, *23*, *24*). In comparison, only a single high-resolution structure of a CDC transmembrane pore has been determined (*18*).

We have recently reported a family of over 300 CDC-like proteins (CDCLs) that span eight different bacterial phyla (*25*). Analysis of the CDCLs belonging to the phylum Bacteroidota revealed the presence of sets of two-component CDCLs, one long CDCL (CDCL^L^) with four domains and one short CDCL (CDCL^S^) lacking the fourth C-terminal domain, that act as a bicomponent complex via a mechanism only previously observed in the mammalian complement MAC (*7*, *26*). A single CDCL^L^ molecule was found to bind to the membrane before the assembly of the pore composed of CDCL^S^ molecules. Unlike CDCs, the CDCLs require proteolytic activation with both components containing a proteolytically cleavable activation loop critical to the regulation of pore formation.

Although the CDCL family were found on the basis of sharing a five-residue sequence motif with CDCs (*25*), their lack of cholesterol dependence, the requirement for proteolytic activation, and the assembly of a heteromeric pore indicated a mechanism of pore formation likely very different from that of CDCs. Here, we present the crystal structure of a proteolytically activated CDCL^L^ and cryo–electron microscopy (cryo-EM) structures of a prepore-like intermediate and the transmembrane pore. Together with biophysical analyses and previously published structures, we can now present structural snapshots across the entire CDCL pore-forming pathway. Our findings provide insights into the conformational changes by which these toxins transition from water-soluble monomers to functional pores and the role of proteolytic activation in the assembly of components to punch holes in the target membrane.

## RESULTS

### Proteolysis results in the loss of the activation loop

We previously reported the crystal structures of the unactivated proforms of CDCL^L^ and CDCL^S^ from the species *Elizabethkingia anophelis* (EaCDCLs, denoted pro-EaCDCL^L^ and pro-EaCDCL^S^, respectively, herein), a member of the Bacteroidota phylum (*25*, *26*). They revealed proteolytic cleavage sites located in loop regions near the N terminus (*25*, *26*). The activation loop in pro-EaCDCL^L^ of 25 residues is located between β strands in domain 2 (D2) (fig. S1A). In contrast, the activation loop of 50 residues in EaCDCL^S^ is substantially longer and extends from the most N-terminal β strand in D2 and winds across the face of domain 3 (D3), where it adds an additional sixth β strand (β0) to the twisted β sheet that is characteristic of MACPF/CDC family members. This β strand then continues into an α helix that sits on top of the domain 1 (D1)-D3 interface before adopting a second β strand in D2 (fig. S1B).

**Fig. 1.**
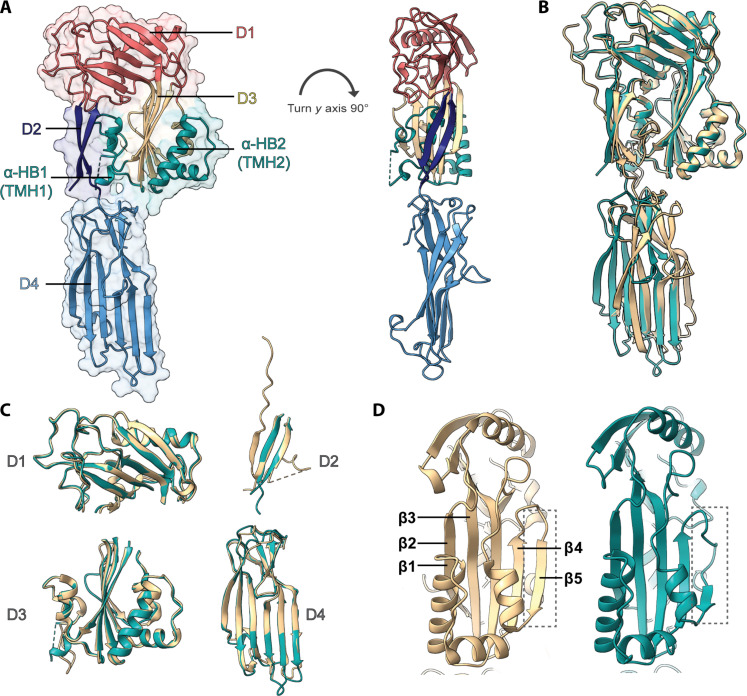
Structural comparison of pro- and act-EaCDCL^L^. (**A**) Crystal structure of act-EaCDCL^L^, solved to 2.49-Å resolution. Left: Shown as a cartoon representation overlaid on transparent surface representation and colored by domain: D1 (pink), D2 (navy), D3 (beige and cyan), D4 (light blue), and α-HBs/TMH regions (cyan). Right: Rotated 90° on the *y* axis and without surface representation. (**B**) Comparison of pro-EaCDCL^L^ crystal structure [Protein Data Bank (PDB) ID: 6XD4] (*25*) (beige) and act-EaCDCL^L^ crystal structure (cyan) superimposed on the D1-D3. (**C**) Structural alignment by domain between the pro-EaCDCL^L^ crystal structure (beige) and act-EaCDCL^L^ crystal structure (cyan). (**D**) Conformational differences in the β5 strand (highlighted by dashed box) in the core D3 β sheet between the pro-EaCDCL^L^ (left) and act-EaCDCL^L^ (right).

The positioning of the activation loop in EaCDCL^S^ is intriguing. Oligomerization of CDCs requires a rotation of the β5 strand away from the β4 strand in the D3 twisted β sheet to allow monomer-monomer interactions between the β4 and β1 strands in adjacent CDC molecules (Fig. 1) (*27*, *28*). This β5 strand forms part of an αβ loop that, in the pore structure of CDCs, adopts a helix-turn-helix (HTH) motif (*18*), a structure also observed in MACPF family members. If the same monomer-monomer interactions were to form between EaCDCL^S^ molecules during oligomerization, then the β strand in the unique insertion region needs to move away to allow access to the β1 strand.

Therefore, we examined how proteolytic activation affected the structures of activated CDCLs. EaCDCL^L^ was activated with proteinase K and purified, yielding the expected cleavage product (~50 kDa; denoted as act-EaCDCL^L^) (*26*), as determined by SDS–polyacrylamide gel electrophoresis (PAGE) and liquid chromatography–mass spectrometry (LC-MS) analysis, and remained monomeric in solution as determined by size exclusion chromatography–multiangle light scattering (SEC-MALS) (fig. S1, C to E). EaCDCL^S^ was activated with trypsin (act-EaCDCL^S^). The purified sample behaved as a monomeric protein but was prone to rapid aggregation on standing at room temperature, but not at 4°C (fig. S2A). Visualization of the aggregated species by negative-stain EM revealed that the aggregant was a stack of circular oligomers (fig. S2B). In contrast, pro-EaCDCL^S^ was not observed to undergo similar oligomerization (fig. S2C). Act-EaCDCL^S^ was confirmed to be cleaved at the known cleavage site (^84^TK^85^) (fig. S1, F and G) (*26*). We confirmed the pore-forming activity of the purified act-EaCDCL proteins using liposome-release assays before structural analysis (fig. S3).

We determined the crystal structure of act-EaCDCL^L^ to 2.49-Å resolution (table S1), revealing the absence of electron density for the D2 β1 strand and its preceeding N-terminal extension (Fig. 1A) (*25*), consistent with the MS results above that the peptide dissociates from the protein. This is despite numerous interactions observed between the β1 strand and the β2 strand in the D2 in the structure of pro-EaCDCL^L^ (fig. S1A). Overll, the crystal structures of pro- and act-EaCDCL^L^ superimpose closely, except for differing orientations of domain 4 (D4) with respect to the rest of the domains (33.5° rotation around *y* axis, shift of 1.4 Å on both *x* and *y* axes) (Fig. 1B). We attribute this to altered crystal packing between the two structures and likely flexibility of the domain interface due to very limited interdomain contacts. Superimposing individual domains showed strong structural similarity (Fig. 1C). One notable difference between the pro- and act-EaCDCL^L^ crystal structures is the slight outward movement (1.2 Å) and shortening of the β5 strand (~15 to ~6.5 Å) in the core D3 β sheet in the activated structure (Fig. 1D and movie S1). Movement of this strand in CDCs is critical for oligomerization and assembly of the prepore state (*29*). The conclusions drawn from the crystal structures are supported by SEC–small-angle x-ray scattering (SAXS) analysis of the proteins in solution (fig. S4 and table S2). Unlike act-EaCDCL^L^, act-EaCDCL^S^ did not crystallize, although SEC-SAXS analysis of EaCDCL^S^ suggested minor differences between the proform and activated states (fig. S5 and table S2).

### Cryo-EM structure of the EaCDCL pore complex

Oligomer formation by CDCLs can be imaged upon lipid monolayers by negative-stain EM, as reported previously (*25*, *26*). We observe similar oligomer formation when using a simple 1-palmitoyl-2-oleoyl-*sn*-glycero-3-phosphocholine (POPC) lipid monolayer or more complicated lipid compositions such as *Escherichia coli* lipid extract (fig. S6). Here, to obtain a higher-resolution structure of the pore complex, we imaged the membrane-inserted EaCDCL pore directly on the surface of POPC liposomes (fig. S7A) and performed cryo-EM single-particle analysis (fig. S8).

Inspection of two-dimensional (2D) class averages did not reveal obvious heterogeneity in the number of subunits per pore complex (fig. S7B)—a problem that has appeared inherent to many other MACPF/CDC pores imaged by cryo-EM [reviewed in (*30*)]. Instead, we observed that complexes were consistently composed of 30 subunits, as previously suggested by biochemical studies (*26*). However, we did observe a high degree of heterogeneity in the number of stacked rings per particle, ranging from a single inserted ring-shaped oligomer (“single-stacked”) up to particles with at least six stacked rings (fig. S7B). We attribute this stacking to the high propensity of act-EaCDCL^S^ to oligomerize at higher temperatures (37°C as used here) even in the absence of lipid, as similar stacked species can be visualized by negative-stain EM for EaCDCL^S^ alone in buffer (fig. S2B). Therefore, we hypothesize that the stacked rings are “prepore-like” structures composed of act-EaCDCL^S^ protein alone, similar to the circular prepore intermediate observed in CDCs (*31*). Similar prepore formation in solution subsequent to proteolytic cleavage of a propeptide has also been observed for some MACPFs, such as Mpf2Ba1 (*6*), and other β-class pore-forming toxins, such as aerolysin (*1*, *32*). However, any putative physiological prepores would be expected to be composed of 1 EaCDCL^L^ molecule and 29 EaCDCL^S^ molecules, similar to the transmembrane pore (*26*), but such a short-lived intermediate would not been captured here because of the long incubation time of the experiment.

We previously reported that functional pores, measured using liposome release assays, require the addition of both EaCDCL^L^ and EaCDCL^S^ and that the stoichiometry of pore formation is 1 EaCDCL^L^ molecule to 29 EaCDCL^S^ molecules (*26*). Using Förster resonance energy transfer and negative-stain imaging with gold-labeled EaCDCL^L^, we previously showed that the single EaCDCL^L^ molecule remains in the final membrane-inserted pore (*26*). We carried out cryo-EM processing without implementing symmetry (C1) to identify a region that would correspond with the EaCDCL^L^ molecule. However, we were unable to unambiguously visualize the EaCDCL^L^ molecule, even after trialing various workflows incorporating symmetry expansion of particles, signal subtraction, focused refinement, 2D/3D classification, and 3D variability analysis of smaller wedges of the complex to extract the asymmetrical features provided by EaCDCL^L^.

**Fig. 2.**
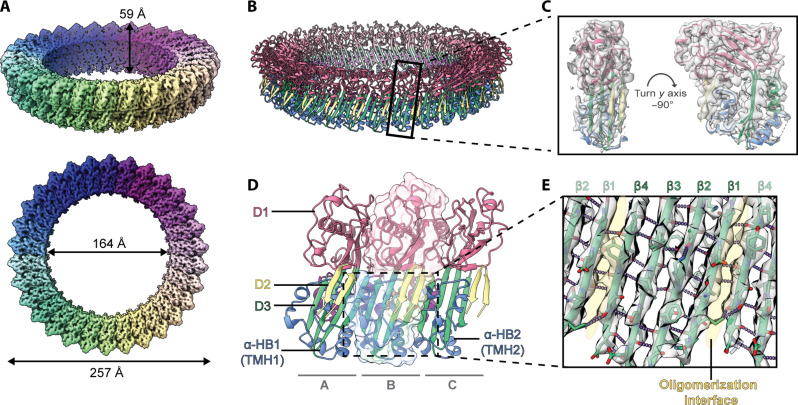
The cryo-EM structure of the prepore-like complex. (**A**) The cryo-EM map at 3.13-Å resolution of the prepore-like structure from focused refinement with C30 symmetry, revealing a circular oligomer with a pore inner diameter of 164 Å and a height of 59 Å. (**B**) Overview of the prepore-like structure built into the cryo-EM map, colored according to domain: D1 (pink), D2 (yellow), D3 (green and blue), and α-HBs (blue). The black box designates a single subunit. (**C**) The fit of the atomic model (single subunit) to the cryo-EM map. (**D**) Three adjacent subunits, shown in cartoon representation and colored as per (B). The middle subunit is shown as a cartoon representation overlaid on transparent surface representation to emphasize subunit boundaries. (**E**) Hydrogen bonds within the core β sheet in D3 stabilize the oligomer. One complete subunit neighbored by two incomplete subunits is shown, with hydrogen bonds shown in purple, the oligomeric interface highlighted by yellow shading, and β strands labeled accordingly.

**Fig. 3.**
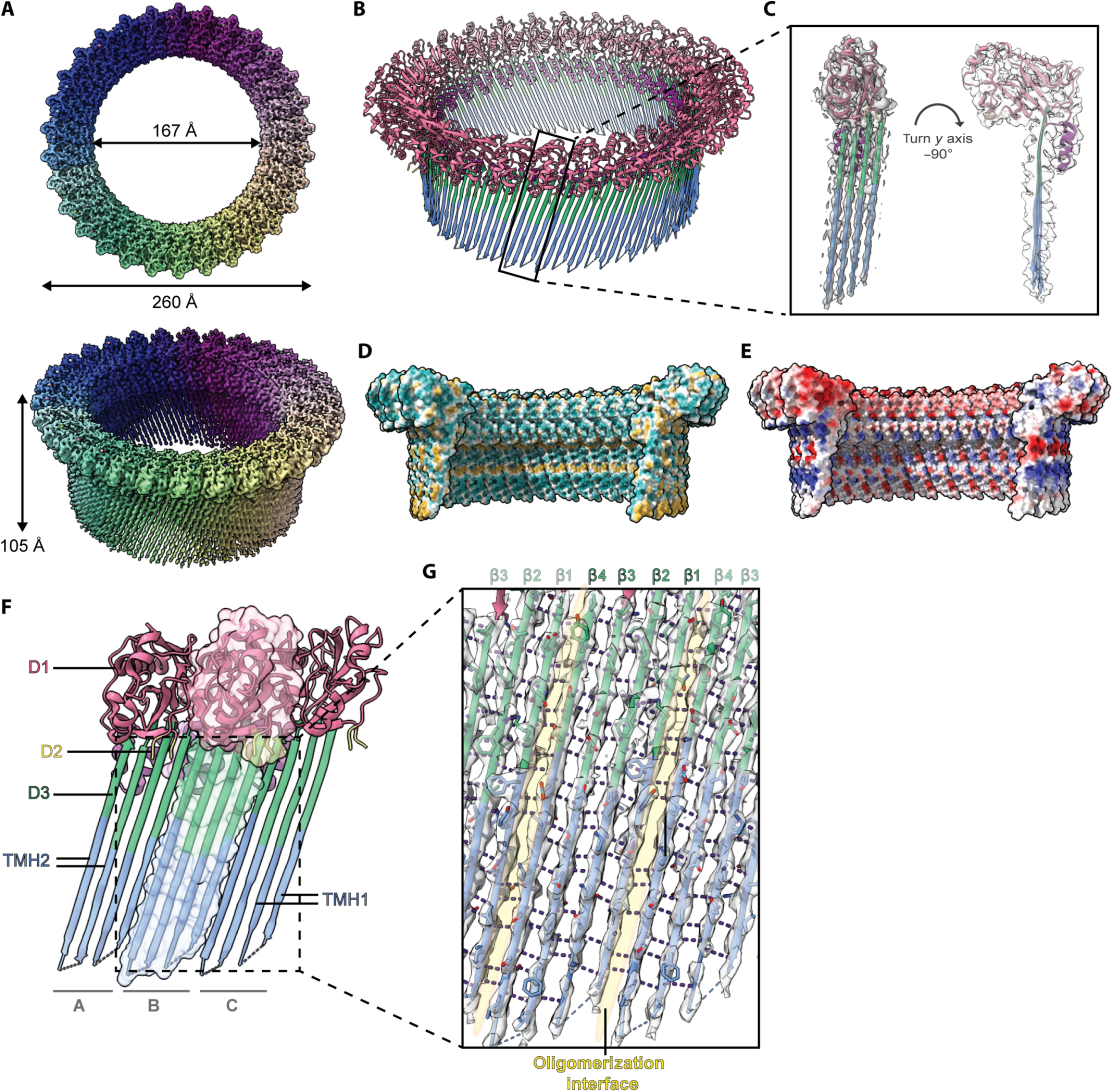
The cryo-EM structure of the EaCDCL inserted pore complex. (**A**) The cryo-EM map at 2.87-Å resolution of the membrane-inserted pore complex, from focused refinement with C30 symmetry, reveals a β barrel pore with an outer diameter of 260 Å, an inner diameter of 167 Å, and a height of 105 Å. (**B**) The pore complex colored according to domain: D1 (pink), D2 (yellow), D3 (green and blue), and α-HBs (blue). The black box designates a single subunit. (**C**) The fit of the atomic model to the cryo-EM map, colored as per (B). (**D**) Surface representation of the transmembrane pore colored according to hydrophobicity (hydrophilic in cyan and hydrophobic in yellow). (**E**) Surface representation of the transmembrane pore colored according to electrostatic potential (negative potential in red and positive potential in blue). (**F**) Three adjacent subunits, shown in cartoon representation and colored as per (B). The middle subunit is shown as a cartoon representation overlaid on transparent surface representation to emphasize subunit boundaries. (**G**) Hydrogen bonds within the core β sheet in D3 stabilize the oligomer. One complete subunit neighbored by two incomplete subunits is shown, with hydrogen bonds shown in black, the oligomeric interface highlighted by yellow shading, and β strands labeled accordingly.

We attribute this to multiple factors. First, the D1-D2-D3 of EaCDCL^L^ and EaCDCL^S^ are remarkably similar in structure [<3-Å root mean square deviation (RMSD)] and would be indistinguishable at low resolution. The ability to distinguish the two is further affected by the large pixel size of the data (1.34 Å per pixel). There is only ~35% sequence identity between the two proteins overall, but inspection of key differing residues in the medium–to–low-resolution C1-processed cryo-EM map revealed somewhat poor side-chain density that was difficult to unambiguously assign. Therefore, we were heavily reliant on the presence of the EaCDCL^L^ D4 domain in our cryo-EM maps to confidently assign the location of EaCDCL^L^. This domain turned out to be missing in the cryo-EM reconstruction, which could be due to the small size of the domain (~19 kDa) and, thus, the overall minimal contribution of this density and corresponding signal with regard to the overall molecule (~1.9% of pore complex and ~0.9% of double-stacked pore-prepore molecule). Furthermore, given that many particles are orientated with a side-view profile (fig. S7B), the single EaCDCL^L^ molecule in the 30–nucleotide oligomer pore was unlikely to be captured in a high proportion of side views. Last, we suspect that D4, connected by a flexible linker to D1-D3, is highly mobile at least in the monomeric state. In our two crystal structures of EaCDCL^L^, D4 was observed in different orientations (Fig. 1), although this flexibility may be reduced by membrane interactions in the pore state. Nevertheless, our previous EM analysis using gold-labeled EaCDCL^L^ showed its presence in the completed pore (*26*). We also refined the final maps with C30 symmetry to yield a higher-resolution map for modeling of the EaCDCL^S^ subunits, as described below (Figs. 2 and 3 and figs. S9 and S10).

### Prepore structure reveals intermediate structural changes

**Fig. 4.**
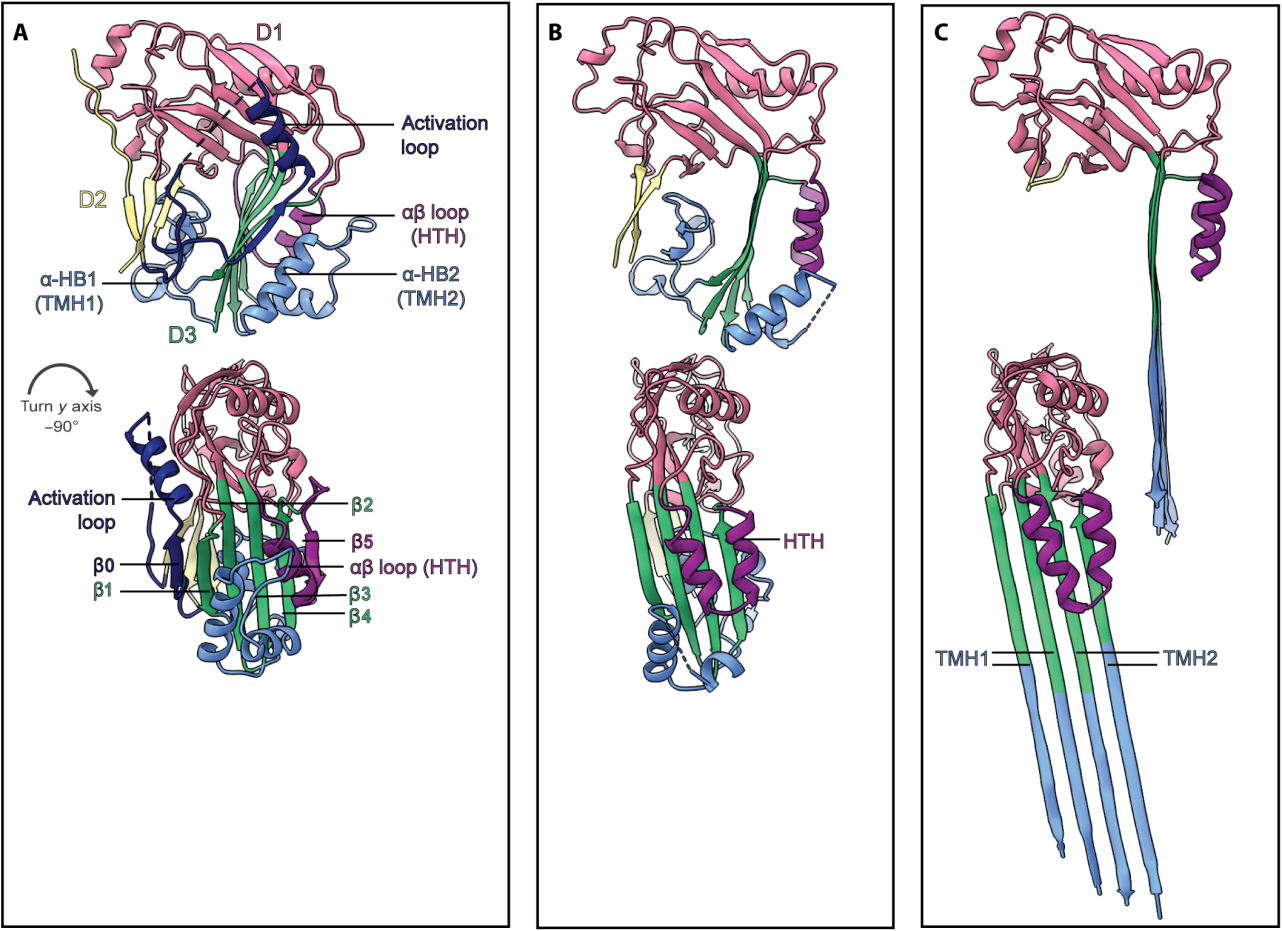
Structure of EaCDCL^S^ in different states along the pore assembly pathway reveals dramatic conformational changes. (**A**) Crystal structure of monomeric pro-EaCDCL^S^ in cartoon representation. Molecules colored according to domain and motif: D1 (pink), D2 (yellow), D3 (green and blue), αβ loop (purple), α-HBs (blue), and activation loop (navy). Bottom molecule is rotated −90° around the *y* axis with respect to the top molecule. (**B**) Single subunit of EaCDCL^S^ from the prepore-like oligomer, colored as per (A), reveals the loss of the activation loop and conversion of the αβ loop to the HTH motif. (**C**) Single subunit of EaCDCL^S^ from the inserted pore complex, revealing complete unfurling of the α-HBs into the TMHs.

Analysis of the cryo-EM map of the prepore-like structure at 3.13-Å resolution (fig. S9B) reveals a large circular oligomer with a total outer diameter of 247 Å, an inner pore diameter of 164 Å, and a height of 59 Å (Fig. 2A). We built an atomic model of the full prepore-like oligomer into the cryo-EM map (Fig. 2B and table S3), which consists of 30 EaCDCL^S^ subunits, each lacking the activation loop and preceding N-terminal region (Figs. 2C and 4B). A comparison of the EaCDCL^S^ protomer in the prepore state with the crystal structure of the monomeric proform of EaCDCL^S^ (Fig. 4, A and B) reveals that while the α-HBs remain in their helical state, the β5 strand of the core D3 β sheet has undergone a complete β-to-α transition to create the HTH motif as observed in the membrane-inserted form of CDCs (*18*). This HTH forms a small α barrel within the interior of the circular oligomer. Thus, loss of the activation loop and corresponding β0 strand, together with the transition of β5, has allowed for the β4 and β1 strands of the core β sheet to form oligomeric contacts between adjacent subunits (Fig. 2, D and E). This corresponds with the canonical oligomeric rearrangement and interactions observed in CDCs (*18*, *29*, *33*).

Comparison of the core β sheet between monomeric EaCDCL^S^ and the prepore-like structure also reveals the loss of the twist observed in the monomeric state, with a slight rotation and straightening of the β1-β4 strands (Fig. 4, A and B). This might be the first step in a transition toward the angled, fully straightened β strands as seen in membrane-inserted CDC pores. In CDCs, the straightening of the core β sheet and disengagement of β5 from the D3-D1/2 interface is mediated by a highly conserved glycine pair. This glycine pair, located within the β turn between the β4 and β1 strands in CDCs, is observed at the same position in EaCDCL^S^ (fig. S1B), suggesting that it plays a similar role in the transition to the prepore observed here.

### Structure of the pore reveals giant β barrel

Analysis of the cryo-EM map for the membrane-inserted pore at 2.87-Å resolution (fig. S10B) reveals a circular complex with the same outer and inner diameter as the prepore structure but with a substantially increased height of ~105 Å due to the full extension of the TMHs (Fig. 3A). We modeled 30 subunits of EaCDCL^S^ into the map (Fig. 3, B and C, and table S3), allowing a complete visualization of the membrane pore complex. This revealed the complete unfurling of the α-HBs to form the membrane-inserted TMHs that make up the β barrel pore, which is primarily hydrophilic and neither strongly positively nor negatively charged in the interior (Fig. 3, D and E). Each EaCDCL^S^ contributes four β strands, at a tilt of ~20 Å to the membrane normal, to yield a β barrel pore composed of 120 β strands stabilized by an extensive network of hydrogen bonds (Fig. 3, F and G). As in the prepore-like oligomer, the HTH forms an interior α barrel, creating the narrowest portion of the pore. Comparison of the EaCDCL^S^ protomer from the pore with the structure from the prepore state reveals no substantial changes beyond the TMH unfurling and further straightening of the core D3 β sheet (Fig. 4, B and C).

**Fig. 5.**
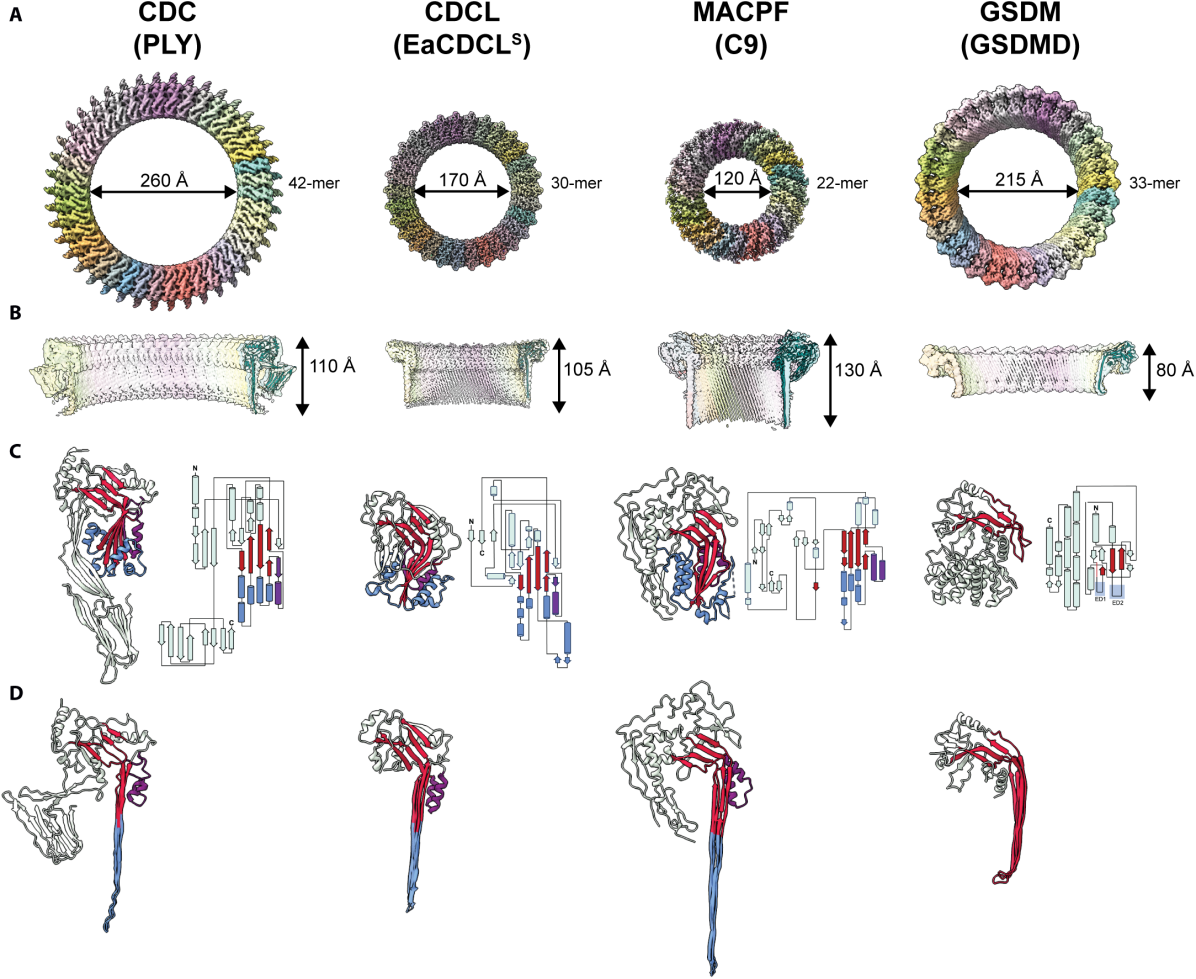
Comparison with the CDC PLY, MACPF C9, and GSDMD. (**A**) The cryo-EM maps of the PLY pore (EMD-4118) (left) (*18*) showing 42 subunits and a pore diameter of 260 Å, the EaCDCL^S^ pore (second from left), showing 30 subunits and a pore diameter of 167 Å, the polyC9 pore (EMD-7773) (second from right) (*8*), showing 22 subunits and a pore diameter of 120 Å, and the GSDMD pore (EMD-21160) (right) (*14*) showing 33 subunits and a pore diameter of 215 Å. All are shown as a top view of the pore and scaled in size with respect to one another. mer, protomer. (**B**) Cryo-EM maps as per (A) represented as a cut through view of the side profile with the fitted model of a single protomeric subunit shown in teal as a cartoon representation: PLY (left) (PDB ID: 5LY6) (*18*), EaCDCL^S^ (second from left), C9 (second from right) (PDB ID: 6DLW) (*8*), and GSDMD (right) (PDB ID: 6VFE) (*14*). (**C**) Crystal structure of monomeric PLY (left) (PDB ID: 5AOD) (*107*), pro-EaCDCL^S^ (second from left), murine C9 (second from right) (PDB ID: 6CXO) (*8*), and GSDMD (right) (PDB ID: 6N9O) (*57*). Shown as a cartoon representation, with core MACPF/CDC β sheet shown in red, α-HB/TMH regions shown in blue, and αβ-loop/HTH region shown in purple. A cartoon topology diagram for each respective structure is shown to the right of each crystal structure. (**D**) Cartoon representation of PLY (left), EaCDCL^S^ (second from left), C9 (second from right), and GSDMD (right) in the pore state, colored as per (C).

As we were unable to resolve EaCDCL^L^ in our cryo-EM reconstructions, we used AlphaFold2 (AF2) (*34*) and the existing structures of pneumolysin (PLY) [Protein Data Bank (PDB) ID: 5LY6] (*18*) and EaCDCL^S^ in the pore state, as well as the D4 of EaCDCL^L^ (PDB ID: 6XD4) (*25*), to obtain two predicted models of EaCDCL^L^ in the pore state at relatively high confidence (fig. S11, A to D). These reveal the canonical MACPF/CDC domain in a nontwisted form with full extension of the TMHs and complete formation of the HTH, as is observed for EaCDCL^S^. The unique D4 is placed at the side of the β hairpins as is the case for PLY (Fig. 5). The two predicted models differ primarily in their placement of D4 with regard to the rest of the molecule (a rotation of ~25° between the two), expected given the predicted flexibility of the interface between D4 and D1-D2-D3 (fig. S11E).

To investigate the feasibility of this model further, we removed a single EaCDCL^S^ subunit from the modeled transmembrane pore and replaced it with the top ranking AF2 EaCDCL^L^ model. After initial refinement in Coot (omitting the D4), the model was subject to refinement in PHENIX (fig. S12, A and B). Overall, the refined model resembles the AF2 models, albeit with minor differences in the D1 domain (Cα RMSD of 0.72 and 0.96 Å to AF2 rank 1 and rank 2 models, respectively) (fig. S12C).

Analysis of the interface between the refined EaCDCL^L^ subunit and adjacent neighboring EaCDCL^S^ subunits in the transmembrane pore revealed numerous putative hydrogen-bonding interactions (55 total) that would stabilize the β barrel structure (fig. S12D). These are formed via the β1 and β4 strands in CDCL^L^, which suggests that the initial movement of β5 observed in the act-EaCDCL^L^ structure (Fig. 1) continues further to form the HTH and expose the β4 strand for oligomerization. These interactions are comparable to the number of interactions formed between EaCDCL^S^ subunits (54 total) (fig. S12E) and, similar to EaCDCL^S^, are equally distributed between the preceding and subsequent neighboring subunits. Overall, this suggests that the presence of a single EaCDCL^L^ subunit in the inserted pore is feasible and not likely to be destabilizing.

We wondered whether the placement of the EaCDCL^L^ D4 in the pore would reveal why CDCL^L^ cannot form the pore alone. Using the refined EaCDCL^L^ model, we modeled a 30–mer pore made up entirely of CDCL^L^ subunits (fig. S12F). No steric clashes between neighboring D4 were observed. However, placement of this model in the context of the cryo-EM maps with full liposome signal (i.e., early reconstructions in the cryo-EM processing workflow, before signal subtraction of the liposome signal) suggest that this conformation may not be compatible within the context of a membrane due to minor steric clashes of D4 with the liposome signal (fig. S12G). This not only questions the placement of D4 in the AF2 models, as also suggested by the Predicted Aligned Error (PAE) plots for these models (fig. S11D), but also correlates with the aforementioned flexibility of D4 that would enable movements that prevent such a steric clash.

The published structure of MAC revealed an asymmetry in the pore manifested by a twisted split-washer configuration causing a break in the β strand packing and, thus, an incomplete ring (*7*, *35*, *36*). A similar configuration has also been observed, although in low abundance, for the MACPF perforin-2 structure (*23*). We investigated the possibility that CDCLs also assemble in similar fashion. We do not see any height difference in the transmembrane pore density, although the lack of top views and very few single-stacked pores in our liposome-based cryo-EM approach limit our opportunity to observe these features. However, we also observe incomplete rings of the prepore-like oligomer when processed with no symmetry (C1 reconstructions) that may not close because of the lack of EaCDCL^L^, be a heterogeneous mix of growing prepore-like oligomers, or result from the transmembrane pore not being perfectly planar, as would be the case if a split-washer structure existed. While we also do not observe a break in the pore density, which may exist with or without height difference, the lack of top views in the cryo-EM again may prevent this observation. Top views are abundant in our negative-stain analysis (*25*, *26*) but do not reveal a disconnect in the ring, although if this disconnect exists and is relatively small, we may lack the resolution to perceive it unambiguously. In contrast, the split-washer effect of the MAC is notable and can be easily observed by negative-stain-EM, cryo-EM and cryo–electron tomography (*7*, *35*, *36*). Thus, further analysis of CDCL pores is required to confirm or rule out such a scenario.

### Structural comparisons with CDC, MACPF, and GSDM family members

To date, the only structure of a fully membrane-inserted CDC pore is for PLY, solved using liposome-extracted pores in amphipol A8-35 (Fig. 5A) (*18*). A major difference between our pore structure and the PLY pore structure is the smooth exterior of the EaCDCL pore, revealing full visualization of the β strands in the β barrel pore. In comparison, the exterior of the PLY pore is studded with D4 that effectively mask the exterior-facing surface of the β barrel (Fig. 5B). Furthermore, the CDCL pore complex presented here is substantially smaller than the published PLY pore and other CDC pores visualized by various methods (*19*, *37*–*47*). CDCs assemble into pores between 35 and 42 subunits, with the PLY pore visualized by cryo-EM comprising 42 subunits with a pore diameter of 260 Å (Fig. 5A). In contrast, the EaCDCL pore contains only 30 subunits with a substantially smaller pore inner diameter of ~167 Å. This pore diameter is consistent with our previous estimates from negative-stain EM analysis (*25*).

The smooth exterior and smaller diameter of the EaCDCL pore are more reminiscent of the polyC9 pore formed in vitro (Fig. 5, A and B) (*8*, *48*)—the primary self-oligomerizing and membrane-spanning component of the MAC pore. However, the CDCL pore is simpler than the polyC9 pore, which is studded by three small accessory domains; the thrombospondin-like domain, low-density lipoprotein receptor A (LDLRA) domain and epidermal growth factor type 2 domain. The LDLRA and the slightly longer TMH regions of C9 result in a pore complex that is ~25 Å taller than that observed for EaCDCL^S^.

Comparison of the monomeric structures for each of these pore complexes reveals the shared, core twisted β sheet and associated α-HBs (or TMH regions). While monomeric PLY and EaCDCL^S^ have an αβ loop yet to undergo the switch to the HTH form, monomeric C9 already has the HTH in its fully helical state (Fig. 5C). Of the three, EaCDCL^S^ is the smallest and simplest molecule, lacking the additional receptor and accessory domains of PLY and C9. Furthermore, structurally and mechanistically, CDCLs seem to straddle both the MACPF and CDC lineages of the superfamily. We hypothesize that the short form of CDCLs may represent a common MACPF/CDC ancestor, from which CDCs, CDCL^L^s, and MACPFs have diverged and gained additional domains.

Comparison of the protomeric conformation of EaCDCL^S^ and PLY in the pore context reveals strong similarities [template modeling (TM) score of 0.841 and DALI *z*-score of 15.2], particularly after omitting domains 2 and 4, which are not observed in the former (Cα RMSD of 1.2 Å). This includes full extension of TMHs of a similar length, with a total of four hairpins per molecule and positioning of the D1. Comparison with the protomeric conformation of C9 revealed similarities to a lesser extent (TM score of 0.576, DALI *z*-score of 10.3), with still lower scores for other MACPF members for which pore structures exist (table S4).

One particularly noteworthy difference between CDCs and CDCLs is the lack of a vertical collapse in EaCDCL^S^ prepore upon pore formation (Fig. 5, C and D). Comparison of the various structures of EaCDCL^S^ presented here reveals that the only change in height is due to unfurling of the α-HBs into the TMHs, while the D1 remains in the same position (Fig. 4). In contrast, a ~40-Å vertical collapse is a key component of the CDC mechanism where, by rotation of the D2, the D3 is brought closer to the target membrane so that when fully unfurled, the TMHs can span the depth of the membrane (*18*, *19*, *37*, *49*). A similar vertical collapse has also been observed for aerolysin and the related family member lysenin (*1*, *2*, *50*) but does not exist for MACPF family members. Such a collapse is necessary for CDCs, given the initial act of membrane binding by the distal tip of the CDC molecule within D4. In contrast, EaCDCL^S^ lacks a D4, instead relying on EaCDCL^L^ for this role, and, therefore, during the initial oligomerization of the complex, the D3 of EaCDCL^S^ is already sufficiently close to the membrane. We postulate that EaCDCL^L^ likely undergoes a collapse similar to CDCs, perhaps before the binding of EaCDCL^S^, as we do not observe a region of the membrane-inserted pore that sits higher than the rest of the complex. While we did not observe a rearrangement of D4 conducive to this collapse in the predicted pore structures of EaCDCL^L^ (fig. S11, A and B), this may appear to reflect the lack of experimental pore-state structures for proteins with this D4, as demonstrated by the PAE plots derived for each model (fig. S11D).

A further family of giant pore β-PFPs is the GSDMs, for which mammalian, fungal, and bacterial family members have been identified (*51*–*56*). Visual inspection of GSDM pores, such as for the mammalian GSDMD (*14*), reveals large β barrels where each subunit contributes two β hairpins per protomer, as is the case with MACPF/CDCs, but which are substantially shorter in height (Fig. 5, A and B). A core twisted β sheet similar to the MACPF/CDC domain is present in GSDMs, although in place of the helical bundles that form the TMHs, there are loops referred to as extension domains 1 and 2 (*13*–*15*). These are commonly not imaged in monomeric GSDM crystal structures such as GSDMD (*57*), but in the pore state, they are substantially shorter and more curved than MACPF/CDC β hairpins, resulting in the shorter transmembrane pore (Fig. 5, C and D).

CDCLs and bGSDMs have several other similarities including the need for proteolytic activation, an activation region being as little as 20 to 40 amino acids (albeit at opposite ends of the molecules), the absence of the vertical collapse characteristic of CDCs in the prepore to pore transition, and the ability to form pores in simple lipid systems. However, there is little detectable amino acid sequence similarity between the families, and the GSDMs lack the conserved diglycine and HTH motifs found in CDCLs. The presence of an evolutionary relationship between the CDC/MACPF and GSDM families has been considered previously but was believed to be unlikely (*15*, *58*–*60*). However, the recent discovery of bGSDMs, including identification of structures in the AF2 database of bGSDM-like proteins with GSDM pore-forming domains fused to immunoglobulin-like β-sandwich domains homologous to the CDC D4, suggests bGSDMs to be the ancestral link (*11*). We observed EaCDCL^S^ to have slightly higher structural similarity to the pore state of bGSDM from *Vitiosangium* sp. (TM score of 0.478 and DALI *z*-score of 8.7) than the most similar mammalian GSDM (TM score of 0.437 and DALI *z*-score of 8.0) (table S4). These fused GSDMs do not have a regulatory protease encoded in their operon unlike other bGSDMs.

## DISCUSSION

We have used x-ray crystallography, SAXS, cryo-EM and biochemical analysis of the short and CDCL^L^ pair from *E. anopheles* to reveal the molecular details of how these water-soluble proteins assemble into the archetype large CDC-like pore in cell membranes. The published crystal structure of pro-EaCDCL^S^ revealed a unique placement of its activation loop, compared to other proteolytically activated members of the wider MACPF/CDC superfamily (*6*, *61*–*65*) or the similar GSDMs (*57*, *66*–*68*), including the recently characterized bGSDM belonging to the same Bacteroidetes phylum as *E. anophelis* (*11*, *54*). Our biochemical and MS analysis revealed proteolytic activation triggered the loss of the activation loop and preceding N terminus from the monomeric EaCDCL^S^ molecule allowing for an oligomerization interface, reminiscent of CDCs, to form between adjacent CDCL molecules.

These observations explain the need for proteolytic activation of CDCL^S^. In CDCs, D4, which, in CDCLs, is either absent or of a completely unrelated sequence, regulates conformational changes in remote D3 regions upon interacting with membrane-bound cholesterol. In the absence of D4 allostery, the CDCL activation loop protects the oligomeric interface from surrounding molecules, a protection that is lost upon cleavage and removal of the activation loop and β0 strand. This also explains the high propensity for aggregation of EaCDCL^S^ following proteolytic cleavage in vitro. The crystal structure of act-EaCDCL^L^ suggests that its activation loop plays a similar role in triggering structural changes that allow for oligomerization, as we observe an initial movement of the β5 strand away from the β4 strand that forms oligomeric interactions in CDCs. However, we are unable to visualize the oligomeric interface of EaCDCL^L^ in the pore complex, although predicted models show a complete β strand–to–α helix transition by the β5 strand to form the HTH and uncover the β4 strand oligomeric interface. Furthermore, the modeling of EaCDCL^L^ in the pore state revealed that the β1 and β4 strands can form substantial interactions with adjacent EaCDCL^S^ subunits within the context of the transmembrane pore.

**Fig. 6.**
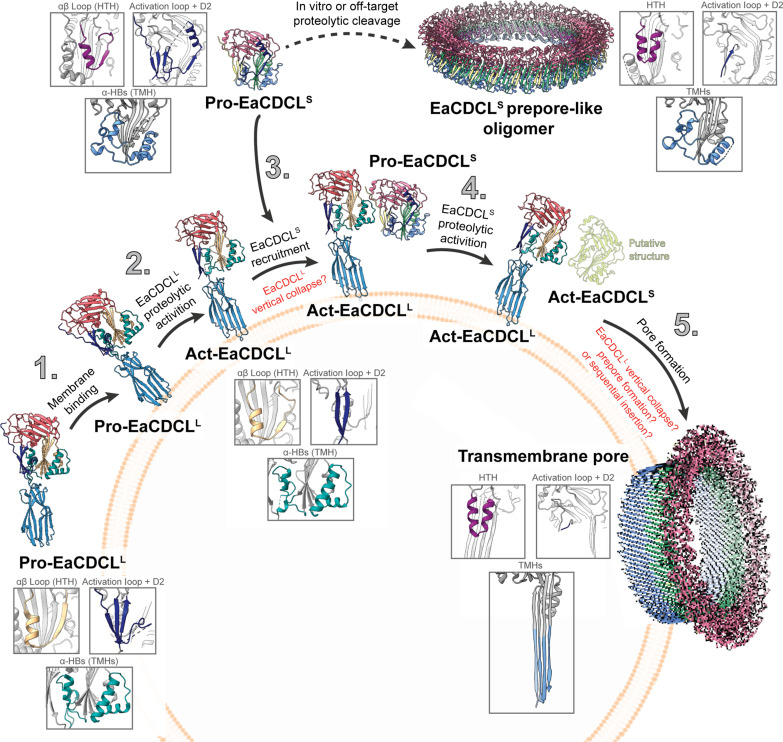
Proposed mechanism for EaCDCL pore formation. Soluble, monomeric pro-EaCDCL^L^ binds to an unknown receptor on the surface of target cells (1) and undergoes proteolytic-cleavage by a membrane-surface protease to form the act-EaCDCL^L^ (2). This form lacks the activation loop and preceding N terminus and displays movement of the β5 strand in comparison to pro-EaCDCL^L^. Soluble, monomeric pro-EaCDCL^S^ is recruited (3) and undergoes proteolytic cleavage to form the act-EaCDCL^S^ (4). This form is predicted to lack the activation loop and preceding N terminus but maintains unfurled α-HBs similar to the prepore-like oligomer of EaCDCL^S^ observed by in vitro proteolytic cleavage of EaCDCL^S^. Last, the α-HBs unfurl to form TMHs that create the transmembrane β barrel pore (*5*) via a currently unknown mechanism.

Together with our previous studies (*25*, *26*), we can now propose a detailed mechanism of action for CDCLs at the molecular level (Fig. 6 and movies S1 and S2). EaCDCL^L^ is secreted as a soluble, monomeric proform with a unique D4 and activation loop. We previously showed that D4 was necessary for CDCL binding via the creation of a chimera wherein the cholesterol binding domain from the CDC perfringolysin O was substituted for the CDCL^L^ D4. Upon proteolytic activation, this chimera, when combined with native CDCL^S^, formed pores on liposome membranes containing cholesterol but not on POPC liposomes lacking cholesterol. However, POPC liposomes lacking cholesterol are readily bound, and pores are formed by complexes containing native CDCL^L^. Thus, this experiment shows that the D4 of CDCL^L^ is necessary for membrane binding and recruiting CDCL^S^ to form the pore. Following binding to the target membrane, we propose that the EaCDCL^L^ is subject to proteolytic cleavage by a membrane-associated protease. Previously, the C11-type protease family was identified as the proteases that activate CDCLs in vivo (*25*, *26*). This yields the act-EaCDCL^L^ lacking the activation loop and preceding N terminus that also displays movement of the β5 strand away from the CDC-like β4 strand oligomerization interface. These structural changes then allow the recruitment of soluble pro-EaCDCL^S^ to EaCDCL^L^ at the membrane surface, which allows for spatial regulation of EaCDCL^S^ proteolytic activation. This mechanism minimizes off-target cleavage of EaCDCL^S^ in solution, which would promote the formation of nonfunctional EaCDCL^S^ oligomers that we refer to as prepore-like oligomers. Although we were able to model a convincing poly-CDCL^L^ prepore, the experimental data suggest that upon EaCDCL^L^ activation, EaCDCL^S^ preferentially binds to EaCDCL^L^. We suggest that the membrane-associated act-EaCDCL^S^ displays structural similarities to those observed in the prepore-like oligomeric form, including the loss of the activation loop and a complete β strand–to–α helix transition of the β5 strand to form the HTH. This frees up the β1 and β4 strands of EaCDCL^S^ to allow for canonical CDC-like oligomerization with additional EaCDCL^S^ monomers. We also demonstrate that pore formation involves the unfurling of the α-HBs in EaCDCL^S^ to form TMHs that create a ~167-Å β barrel pore with the entire mechanism lacking a vertical collapse in EaCDCL^S^, as seen in CDCs.

CDCLs, MACPF/CDC, and GSDM families all share a requirement for critical regulatory mechanisms controlling the spatial and temporal formation of their β barrel pores. This ensures that pore formation does not occur spontaneously before binding to the target membrane, but instead biases oligomerization toward the generation of productive pores. However, the finer details of these mechanisms differ substantially between these different families. CDC regulation involves cholesterol recognition by D4 and, in some cases, the receptor CD59, with associated allosteric regulation of D3 conformational changes, including activating the switch that allows the inhibitory β5 strand to dissociate from the oligomeric interface (*27*–*29*, *69*–*72*). Although MACPF family members have more diverse regulatory mechanisms, one of the most complicated systems is exemplified by the complement MAC pore. Regulatory requirements include proteolytic activation of C5, which must first bind to the target cell for efficient activation of the C5 component to initiate formation of the C5b8 complex, which then recruits the pore-forming C9 to form the MAC pore (*7*, *73*–*75*). In contrast, GSDMs are primarily regulated by proteolytic cleavage of an inhibitory C-terminal domain of varying length (*11*, *57*, *66*–*68*, *76*). The recent discovery of bGSDMs led to the identification of structures in the AF2 database of bGSDM-like proteins with GSDM pore-forming domains fused to immunoglobulin-like β-sandwich domains. Since these domains are homologous to the CDC D4, it was suggested that bGSDMs are an ancestral link to CDCs (*11*). We observed EaCDCL^S^ to have slightly higher structural similarity to the pore state of bGSDM from *Vitiosangium* sp. (TM score of 0.478 and DALI *z*-score of 8.7) than the most similar mammalian GSDM (TM score of 0.437 and DALI *z*-score of 8.0) (table S4). Similar to the MAC, CDCLs have multiple levels of regulation that are not shared with CDCs, MACPFs, or GSDMs alone. This includes proteolytic activation of an N-terminal inhibitory loop, restriction of membrane binding to a single component (CDCL^L^) that recruits the pore-forming C9-like CDCL^S^ of the complex, and the required movement of the β5 strand necessary for CDC pore formation. Furthermore, for some CDCLs, we have identified a membrane-bound inhibitory molecule that mimics the role of CD59 for the MAC (*26*).

Future work is needed to address how EaCDCL^L^ interacts with EaCDCL^S^, which includes visualization of EaCDCL^L^ in the pore complex. Studies on how domain D4 in EaCDCL^L^ recognizes lipids and possibly protein receptors are also needed. An ultimate goal would be to understand the detailed molecular choreography between the activating protease, EaCDCL^L^, and EaCDCL^S^ in which the correct order of steps is essential for producing a productive pore.

Our structural analysis presented here, together with our previously reported structural and biochemical data (*25*, *26*), supports the hypothesis that while these pore-forming toxins are named CDC-like proteins and share some similarities with CDCs, they have multiple structural and mechanistic details that fully place them in their own separate, unique family. This includes a requirement for proteolytic activity, two-partner pore formation using a single membrane-binding component, a lack of vertical collapse for pore formation, and targeting of bacteria over eukaryotic cells. In some respects, they mimic the assembly of the MAC pore complex more closely than CDCs.

## MATERIALS AND METHODS

### Expression of EaCDCL^L^ and EaCDCL^S^

Plasmids containing the gene for EaCDCL^L^ or EaCDCL^S^ were transformed into *E. coli* BL21(DE3) competent cells for expression. Cells were cultured in 2-liter flasks of LB medium supplemented with ampicillin (100 μg/ml) at 37°C until an optical density at 600 nm of 0.6 was reached. For EaCDCL^L^, protein expression was induced with isopropyl-β-d-thiogalactopyranoside (IPTG) (0.4 mM) and allowed to proceed at 37°C for 4 hours. For EaCDCL^S^, protein expression was induced with IPTG (0.4 mM) and allowed to proceed at 20°C for 24 hours. Cells were harvested by centrifugation, and cell pellets were stored at −80°C.

### Purification of pro-EaCDCL^L^

EaCDCL^L^ cell pellets were resuspended in lysis buffer [50 mM Hepes (pH 7.0), 500 mM NaCl, 20 mM imidazole, 1 mM tris(2-carboxyethyl)phosphine (TCEP), 5% (v/v) glycerol, and 0.01% (v/v) Triton X-100]. Cells were homogenized using an Emulsiflex C3 high-pressure homogenizer (Avestin), and the lysate was loaded onto a 5-ml HisTrap HP column (Cytiva) preequilibrated in buffer A [50 mM Hepes (pH 7.0), 500 mM NaCl, 20 mM imidazole, and 5% (v/v) glycerol]. Columns were washed with buffer A before bound protein was eluted with an imidazole gradient using buffer B [50 mM Hepes (pH 7.0), 500 mM NaCl, 500 mM imidazole, and 5% (v/v) glycerol]. Fractions corresponding to His-EaCDCL^L^ were pooled and buffer exchanged into 20 mM Hepes (pH 7.0), 150 mM NaCl, 5 mM CaCl_2_, and 5% (v/v) glycerol using a HiPrep 26/10 desalting column (Cytiva). His-EaCDCL^L^ was subject to His-tag cleavage with thrombin overnight at 4°C before cleaved EaCDCL^L^ was further purified by a secondary Ni^2+^–immobilized metal affinity chromatography (IMAC) step. Pooled EaCDCL^L^ was further purified using a HiLoad 16/600 Superdex 200 pg column (Cytiva) equilibrated in 20 mM Hepes (pH 7.0), 50 mM NaCl, 1 mM TCEP, and 5% (v/v) glycerol. Fractions were analyzed by SDS-PAGE, pooled, and stored in aliquots at −80°C.

### Generation of act-EaCDCL^L^

EaCDCL^L^ activation was achieved using proteinase K, as small-scale optimization revealed that this yielded a cleaner cleavage than trypsin, the other protease known to activate the protein. Purified pro-EaCDCL^L^ was cleaved with proteinase K (New England Biolabs) at a ratio of 1:300 (w/w) for 5 min at room temperature before the reaction was stopped with 1 mM phenylmethylsulfonyl fluoride (PMSF). The cleaved sample was purified using a HiLoad S200 16/600 column (Cytiva) equilibrated in 20 mM Hepes (pH 7.0), 50 mM NaCl, 1 mM TCEP, and 5% (v/v) glycerol. Fractions corresponding to act-EaCDCL^L^ were pooled and stored at −80°C. For crystallization, the protein was dialyzed into 20 mM sodium citrate (pH 6.5) and 150 mM NaCl, concentrated to 7.75 mg/ml, and aliquots were stored at −80°C.

### Purification of pro-EaCDCL^S^

EaCDCL^S^ cell pellets were resuspended in lysis buffer [50 mM tris (pH 8.5), 500 mM NaCl, 20 mM imidazole, 2 mM CaCl_2_, 1 mM TCEP, 5% (v/v) polyethylene glycol 400 (PEG 400), and 0.01% (v/v) Triton X-100]. Cells were homogenized using an Emulsiflex C3 high-pressure homogenizer (Avestin), and the lysate was loaded onto two 5-ml HisTrap HP columns (Cytiva) preequilibrated in buffer A [50 mM tris (pH 8.5), 500 mM NaCl, 20 mM imidazole, 1 mM TCEP, and 5% (v/v) glycerol]. Columns were washed with buffer A before bound protein was eluted with an imidazole gradient using buffer B [50 mM tris (pH 8.5), 500 mM NaCl, 500 mM imidazole, 1 mM TCEP, and 5% (v/v) glycerol]. Fractions corresponding to His-EaCDCL^S^ were pooled, and buffer was exchanged into 50 mM tris (pH 8.5), 150 mM NaCl, 5 mM CaCl_2_, 1 mM TCEP, and 5% (v/v) PEG 400 using a HiPrep 26/10 desalting column (Cytiva). His-EaCDCL^S^ was subject to His-tag cleavage with thrombin overnight at 4°C before cleaved EaCDCL^S^ was further purified by a secondary Ni^2+^-IMAC step. Pooled EaCDCL^S^ was further purified using a HiLoad 16/600 Superdex 75 pg column (Cytiva) equilibrated in 20 mM Hepes (pH 7.5), 150 mM NaCl, 2 mM CaCl_2_, and 5% (v/v) PEG 400. Fractions were analyzed by SDS-PAGE, pooled, and stored in aliquots at −80°C.

### Generation of act-EaCDCL^S^

Small-scale optimization revealed trypsin to be the superior choice over proteinase K to achieve the cleanest, fully cleaved sample. Purified pro-EaCDCL^S^ was cleaved with trypsin at a ratio of 1:100 (w/w) for 5 min at room temperature before the reaction was stopped with 1 mM PMSF. The cleaved sample was purified using a HiLoad S75 16/600 column (Cytiva) equilibrated in 20 mM Hepes (pH 7.5), 150 mM NaCl, 2 mM CaCl_2_, and 5% (v/v) PEG 400. Dynamic light scattering (DLS) revealed that if the protein sat at room temperature for as little as 15 min, then it started to aggregate and the hydrodynamic size of the protein slowly increased if incubated for longer at room temperature or at 37°C (fig. S3A). Thus, fractions corresponding to act-EaCDCL^S^ were pooled and stored at −80°C. Analysis by DLS and EM showed that maintaining the act-EaCDCL^S^ protein on ice or at 4°C during experimental analysis at all times prevented aggregation, with pro-EaCDCL^S^ not observed to undergo similar oligomerization (fig. S3, B and D).

### Crystallization of act-EaCDCL^L^

Crystallization trays were set up using the sitting drop method in 96-well Rigaku ultraviolet (UV)+ crystallization plates (Rigaku) using a Gryphon liquid dispensing robot. Crystallization drops were set up using 0.2 μl of act-EaCDCL^L^ (7.75 mg/ml) and 0.2 μl of reservoir solution [0.2 M calcium acetate hydrate and 0.1 M sodium cacodylate: HCl (pH 6.5) and 40% (v/v) PEG 300], with 45 μl of crystallization condition in the reservoir, and incubated at 20°C. Crystals were briefly soaked in cryoprotectant solution [reservoir solution with 20% (v/v) glycerol] before cryocooling in liquid nitrogen.

### Crystal structure determination of act-EaCDCL^L^

X-ray diffraction data were collected at the MX2 Beamline at the Australian Synchrotron at 100 K and a wavelength of 0.9537 Å using the using the ACRF Eiger 16 M Detector. Act-EaCDCL^L^ crystals diffracted to a maximum resolution of 2.49 Å. Diffraction data were processed with XDS (*77*) and AIMLESS (*78*) from the CCP4 software suite, using the ccp4i2 interface (*79*). Crystals belonged to the space group *I*2 with unit cell dimensions of *a* = 61.7 Å, *b* = 24.5 Å, *c* = 300.2 Å, and *b* = 93.2°.

The structure was determined by molecular replacement using Phaser (*80*), using the D1-D2-D3 and the D4 of the published pro-EaCDCL^L^ crystal structure (PDB ID: 6XD4) (*25*) as separate rigid-body search models. Briefly, the initial molecular replacement trial was conducted using a search model consisting of D1, D2, and D3 of the pro-EaCDCL^L^ crystal structure (PDB ID: 6XD4, residues 34 to 367). The output was subject to a second trial using D4 of the pro-EaCDCL^L^ crystal structure (PDB ID: 6XD4, residues 368 to 516). The model was refined by iterative rounds of manual modification in Coot (*81*) and refinement with Phenix.Refine (*82*). The final model geometry was analyzed using MolProbity (*83*).

### LC-MS analysis of intact protein

The molecular mass of purified recombinant proteins was confirmed by LC quadrupole time-of-flight MS (LC-QTOF-MS) analysis. Approximately 5 μg of protein was injected onto a Sepax Bio-C4 (5 μm, 2.1 mm by 50 mm) in-line reversed-phase column (Sepax Technologies) connected to a high-performance LC (HPLC) system (Agilent). The column was washed with buffer A [0.1% (v/v) formic acid and 5% (v/v) acetonitrile in water] before the protein was applied to the column. The applied protein was eluted with a linear gradient of buffer B [0.1% (v/v) formic acid and 80% (v/v) acetonitrile]. MS data were collected using a QTOF (Agilent 6520) in the Bio21 Mass Spectrometry and Proteomics Facility. Data were analyzed using the MassHunter Qualitative Analysis software (Agilent) and GraphPad Prism v.9.4.1 (GraphPad Software, Boston, MA, USA; www.graphpad.com).

### Dynamic light scattering

DLS was performed to assess purified protein samples for aggregation. Protein samples at approximately 2 to 3 mg/ml were dispensed into a low-volume disposable plastic microcuvette (Malvern Panalytical). DLS measurements were performed in triplicate using the Malvern Zetasizer Nano ZS (Malvern Panalytical) at 4°, 25°, or 37°C. Data analysis was performed using Zetasizer software version 7.02 (Malvern Panalytical) and GraphPad Prism v.9.4.1.

### Size exclusion chromatography–multiangle light scattering

SEC-MALS experiments were performed to assess the oligomeric state and molar mass of pro- and act-EaCDCL^L^ in solution. Experiments were conducted using a LC-20 AD HPLC system (Shimadzu), equipped with autosampler and SPD-20C UV-visible detector, coupled to a DAWN NEON 18-angle light scattering detector (Wyatt Technology) with inline quasi-elastic light scattering (QELS) DLS (Wyatt Technology) and an Optilab TrEX refractive index monitor (Wyatt Technology). Pro-EaCDCL^L^ or act-EaCDCL^L^ (both 5.0 mg/ml, 5 μl) protein was injected onto a Superdex 200 Increase 5/150 GL column (Cytiva) equilibrated in 20 mM Hepes (pH 7.5), 150 mM NaCl, 1 mM TCEP, and 0.1% sodium azide. Samples were separated at a flow rate of 0.35 ml/min. Data processing was performed using the Astra software v.7.3.2 (Wyatt Technology), with the refractive increment (dn/dc) set at 0.185 (*84*), and GraphPad Prism v.9.4.1. Calibrations were performed with bovine serum albumin before analyzing experimental samples.

### Small angle x-ray scattering

Scattering data were collected on the SAXS/wide-angle x-ray scattering (WAXS) beamline at the Australian Synchrotron (Clayton, Victoria) using the in-line SEC system with a coupled coflow sample sheath flow environment to reduce radiation damage (*85*, *86*). Purified pro-EaCDCL^L^ (6.0 mg/ml) or act-EaCDCL^L^ (5.0 mg/ml) protein samples in 20 mM Hepes (pH 7.5), 150 mM NaCl, 1 mM TCEP, and 0.1% sodium azide were injected (50 ml) onto a Superose 6 Increase 5/150 GL column (Cytiva) (pro-EaCDCL^L^) or a Superdex 200 Increase 5/150 GL column (Cytiva) (act-EaCDCL^L^) preequilibrated in matching buffer. Purified pro-EaCDCL^S^ (5.0 mg/ml) or act-EaCDCL^S^ (3.5 mg/ml) protein samples in 20 mM Hepes (pH 7.5), 150 mM NaCl, 2 mM CaCl_2_, and 0.1% sodium azide were injected (50 ml) onto a Superdex 75 Increase 5/150 GL column (Cytiva) preequilibrated in matching buffer. Samples were eluted at a flow rate of 0.35 ml/min directly into a 1.5-mm-diameter quartz capillary for data collection. SAXS images were continuously acquired at 1-s exposure at an energy of 11.5 keV (maximum flux of 8 × 10^12^ photons/s) using a Pilatus 2 M detector positioned at a distance of ~2500 mm.

Data reduction of SAXS data was performed using the beamline software ScatterBrain 2.83 (http://archive.synchrotron.org.au/aussyncbeamlines/saxswaxs/software-saxswaxs). Buffer subtraction and selection of frames from the chromatographic peak with a consistent radius of gyration (*R*_g_) was conducted using CHROMIXS (*87*). Analysis of SEC-SAXS data was performed using programs from the ATSAS 3.0.1 program suite (*88*) and plotted using and GraphPad Prism v.9.4.1. SAXS profiles were analyzed with PRIMUS (*89*) and *P*(*r*) distributions obtained using GNOM (*89*). Ab initio bead models were calculated using DAMMIF (*90*) and subsequently aligned and averaged using DAMAVER (*91*), and the averaged model was refined using DAMMIN (*92*). Superimposition of the SAXS ab initio models and crystal structures was conducted using SUPCOMB (*93*). Theoretical scattering from the aforementioned crystal structures was generated and fit to experimental scattering data using CRYSOL (*94*).

### Liposome preparation

Liposomes were composed of POPC (Sigma-Aldrich), which was shown previously to be sufficient for CDCL pore formation (*26*). Lipid was solubilized in chloroform before being dried under nitrogen with continuous rotation to form a thin lipid film. Lipid films were resuspended in buffer [Hepes-buffered saline (HBS), pH 7.4] by vortexing, and lipid mixtures were exposed to 5 cycles of freeze-thawing, where mixtures were snap frozen in liquid nitrogen before being thawed in a warmed (~37°C) ultrasonic water bath with continuous sonication. Lipid suspensions were extruded to generate a uniform solution of unilamellar liposomes using a Mini Extruder kit (Avanti Polar Lipids) with a polycarbonate membrane with a pore size of 0.10 μm. For cryo-EM, liposomes were further extruded using a 0.05-μm membrane. Extruded liposomes were stored at 4°C and used within 2 days.

### Liposome release assays

Liposomes for carboxyfluorescein-release assays were prepared as above, with the addition of 50 mM 6-carboxyfluorescein (CF) into the resuspension buffer. Extruded CF liposomes were subject to buffer exchange using NAP-5 columns (Cytiva) preequilibrated in CF-free HBS (pH 7.4). Liposome-release measurements were conducted in a 96-well plate in triplicate. EaCDCL^L^ protein and CF liposomes (1:200 dilution) were added to each well and measurements initiated at 37°C in a CLARIOstar plus plate reader. After 5 min, EaCDCL^S^ was added to yield a final concentration of 1 μM and molar ratio of 1:1 EaCDCL^S^:EaCDCL^L^. Measurements were continued for a further ~30 min. Data analysis was performed using GraphPad Prism v.9.4.1.

### Negative-stain EM

Carbon-coated copper grids (square mesh, 300 mesh) were glow discharged using a GloQube Plus glow discharge unit (Quorum Technologies Ltd.) for 30 s at 15-mA voltage. For analysis of act-EaCDCL^S^ in solution, act-EaCDCL^s^ (0.6 μM) was incubated at 37°C for 20 min, and 4 μl was applied to the glow-discharged grid for 1 min before blotting using Whatman filter paper. The grid was washed with ultrapure water, followed by staining using 2% (w/v) uranyl acetate (J.T. Baker Chemical Company).

To prepare lipid monolayers for visualizing EaCDCLs on POPC and *E. coli* lipid membranes, a custom Teflon block was used. Act-EaCDCL^L^ (0.3 μM), act-EaCDCL^S^ (0.3 μM), or both act-EaCDCL^L^ and act-EaCDCL^S^ (0.15 and 0.3 μM, respectively) in HBS (pH 7.4) were added to the wells of the prechilled Teflon block. A lipid solution (1 μl, 0.5 mg/ml) composed of POPC (Sigma-Aldrich) or *E. coli* lipid total extract (Avanti Polar Lipids) dissolved in chloroform was placed on top of the wells. The evaporated chloroform allows the formation of the lipid monolayer, on top of which a nonglow-discharged carbon/formvar-coated (square mesh, 300 mesh) grid was placed. The Teflon block was allowed to incubate at 37°C in a humid environment for 15 to 20 min before grids were removed, washed with buffer, and stained as described above.

The grids were imaged at a nominal magnification of ×57,000 and 1.5 to 2.0 μm underfocus with a CETA 4000 by 4000 complementary metal-oxide semiconductor camera, using a Talos L120C 120-kV microscope (Thermo Fisher Scientific) located in the Ian Holmes Imaging Centre at Bio21 Institute.

### Cryo-EM sample preparation and data collection

Act-EaCDCL^L^ and act-EaCDCL^S^ (1:2 molar ratio) were added to liposomes to yield a final sample with a liposome concentration of 3.95 mM and a 1:500 protein:lipid molar ratio. While we have previously shown that the stoichiometry of EaCDCL^L^ to EaCDCL^S^ is 1:29 in the pore complex (*26*), to limit off-target CDCL^S^ oligomers and to avoid any potential bias toward the stoichiometry observed, we obtained cryo-EM data from liposomes incubated with a ratio of 1:2 EaCDCL^L^:EaCDCL^S^.

EaCDCL protein was added sequentially, where act-EaCDCL^L^ (final concentration of 3.95 μM) was added first and allowed to incubate at 37°C for 5 min before the addition of act-EaCDCL^S^ (7.9 μM). The sample was incubated 37°C for a further 15 to 20 min before immediately being applied to Quantifoil R 2/2 UT 300 mesh gold grids, glow-discharged using a PELCO easiGlow cleaning system (Ted Pella) for 20 s at 15 mA. Vitrified cryo-EM grids were prepared using a Vitrobot Mark IV automated vitrification system (Thermo Fisher Scientific) set at 100% humidity and 22°C. Proteoliposome solutions (4 μl) were applied to a freshly glow discharged grid, incubated for 30 s, and blotted for 6 s before plunge freezing. Samples were stored under liquid nitrogen until data collection.

Screening of vitrified cryo-EM grids was conducted using a Talos L120C 120-kV microscope. Data were collected on a Titan Krios (Thermo Fisher Scientific) operating at 300 kV equipped with a K3 (Gatan) direct electron detector with an energy filter (20 eV), located in the Ian Holmes Imaging Centre at Bio21 Institute. Grids were imaged at a nominal magnification of ×64,000 (pixel size of 1.32 Å) calibrated using the crystal lattice spacing from transmission EM images of an oriented gold film.

### Cryo-EM image processing and analysis

Motion correction of raw movies (15,971) was conducted using MotionCor2 (*95*) within RELION-3.1 (*96*, *97*). Contrast transfer function (CTF) estimation was performed within CryoSPARC v4.0 (Structura Biotechnology) (*98*), and poor micrographs were rejected (CTF fit > 5 Å, defocus > 35,000 Å, and astigmatism > 500 Å). A subset of 500 micrographs were subject to manual picking with particles used to train a Topaz model (*99*), which was subsequently use to pick 559,234 particles from 14,978 micrographs. Three rounds of 2D classification were conducted, yielding 326,528 particles.

Most particles were double stacked with these particles (155,216 particles) subject to further processing. After 3D classification, 153,018 particles were used for nonuniform refinement using C1 symmetry in CryoSPARC (*100*). A mask of the lipid bilayer from the liposome was created using the segmentation tool Segger (*101*) in UCSF Chimera v1.16 (*102*) and used for signal subtraction in cryoSPARC, followed by local refinement. This process was repeated to remove further signal of the lipid bilayer before nonuniform refinement with CTF refinement. Heterogeneous refinement was used to remove poorly subtracted particles [124,375 (81.8%) particles kept], duplicate particles were removed (1095 particles removed), and remaining particles (123,280 particles) were subject to a final round of nonuniform refinement using C1 symmetry to yield a map of the double-stacked pore at an overall resolution of 3.35 Å [Fourier shell correlation (FSC) = 1.43]. A further map refined using C30 symmetry was also generated from the C1 map, yielding a map of the double-stacked pore at an overall resolution of 2.97 Å (FSC = 1.43).

The double-stacked map reveals an assumed prepore-like structure stacked upon an inserted pore. To generate separate maps for the prepore and the pore, the double-stacked map (C1, 3.35 Å) was used. For the pore, a map of the prepore was generated using Segger and used for particle subtraction, followed by local refinement of the pore using C1 symmetry to yield a map at a resolution of 3.45 Å (FSC = 1.43). This C1 map was also used to refine using C30 symmetry, yielding a map of the inserted pore with a resolution of 2.87 Å (FSC = 1.43). A similar process was used for the prepore, although this required an intermediate 3D classification step to remove poorly subtracted particles. The remaining 91,117 particles were used for focused refinement of the prepore using C1 symmetry, yielding a map with a resolution of 3.52 Å (FSC = 1.43). Efforts to improve poor regions in the map, likely due to heterogeneity in full completion of the circular prepore, were trialed using further 2D classification (fig. S13). However, this did not improve this region of the map substantially and decreased the quality and resolution of the map overall, as further particles were rejected from refinement. A further map of the prepore was also refined using C30 symmetry, generated from the final C1 map, yielding a map of the prepore with a resolution of 3.13 Å (FSC = 1.43).

To validate the pore structure from the double-stacked map, particles representing only the inserted pore (9533) were subject to ab initio modeling and nonuniform refinement. Following particle subtraction of the lipid bilayer followed by local refinement, nonuniform refinement with CTF refinement and C1 symmetry was performed to yield a map with a resolution of 6.50 Å (FSC = 1.43). A further map refined using C30 symmetry was also generated from the C1 map, yielding a map of the inserted pore with a resolution of 3.44 Å (FSC = 1.43). This confirmed the presence of the same structural features seen in the double-stacked particles described above. All maps have been deposited in the Electron Microscopy Data Bank (EMDB), with accession codes shown in table S3.

### Model building and analysis

Model building of both the prepore-like oligomer and the membrane-inserted pore was conducted using a similar approach. The crystal structure of monomeric pro-EaCDCL^S^ (chain A only) was rigid-body docked into a single subunit of the map in ChimeraX (*103*). The structure was subject to extensive refitting and remodeling, on account of the large conformational changes between monomer and oligomeric states, using Coot (*81*) with real-space refinement performed in PHENIX, before using the modified subunit structure to construct the complete 30–nucleotide oligomer complex models. Models were subject to further inspection and refinement in Coot and PHENIX. Final models were validated using PHENIX, MolProbity (*83*), and the wwPDB OneDep validation server, with validation statistics stated in table S3. Visualization and figure generation were conducted in Chimera (*102*) and ChimeraX (*103*). Pair-wise structural alignment of the EaCDCL^S^ pore model to existing CDC, MACPF, or GSDM pore structures was conducted using the DALI server (*104*) and TM-align (*105*). All models have been deposited in the PDB under accession codes 9CCQ for the EaCDCL^S^ prepore-like oligomer and 9CCP for the EaCDCL^S^ inserted pore.

### Modeling of EaCDCL^L^ in the pore state

Predicted models of EaCDCL^L^ were obtained using AF2 (*34*) via the ColabFold notebook (*106*). The sequence of act-EaCDCL^L^ (residues 77 to 516, lacking the activation loop) was provided as input alongside existing structures of the pore form of PLY (PDB ID: 5LY6) (*18*) and EaCDCL^S^ and the D4 domain of EaCDCL^L^ (PDB ID: 6XD4). Five structural models were generated, with only the top two ranked models having high confidence and complete secondary structure. Models were visualized in ChimeraX.
