# Supplementary material for: Structural basis for the pore-forming activity of a complement-like toxin
Source: Sci Adv. 2025 Mar 28;11(13):eadt2127. doi: 10.1126/sciadv.adt2127 (PMC11952106; doi:10.1126/sciadv.adt2127)
Supplement: Supplementary file 1 — Figs. S1 to S13 Tables S1 to S4 Legends for movies S1 and S2 [file sciadv.adt2127_sm.pdf]

Science Advances
AAAS

Supplementary Materials for

Structural basis for the pore-forming activity of a complement-like toxin

Bronte A. Johnstone *et al.*

Corresponding author: Michael W. Parker, mwp@unimelb.edu.au

Sci. Adv. **11**, eadt2127 (2025)
DOI: 10.1126/sciadv.adt2127

The PDF file includes:

Figs. S1 to S13
Tables S1 to S4
Legends for movies S1 and S2

Other Supplementary Material for this manuscript includes the following:

Movies S1 and S2

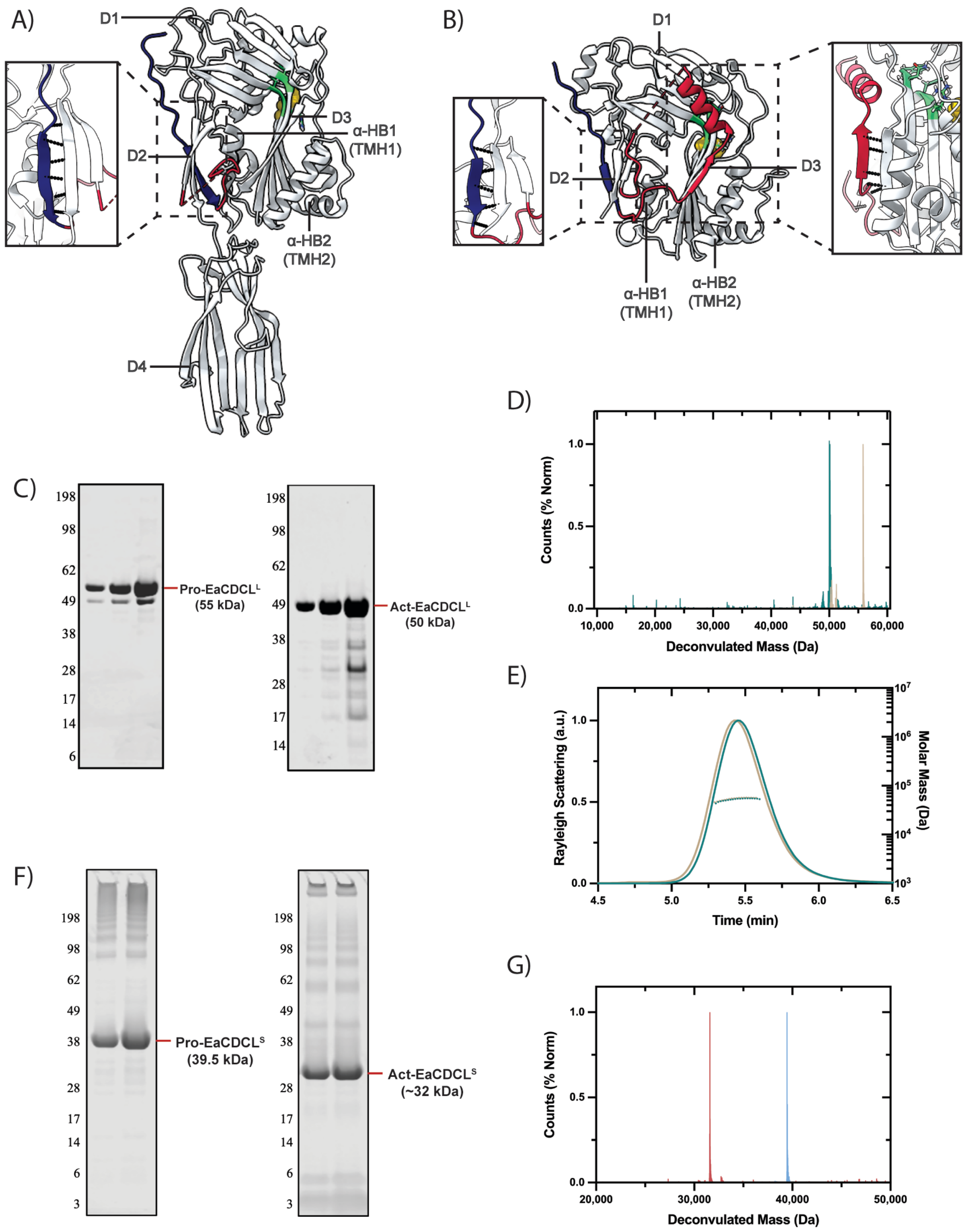

Fig. S1: Proteolytic cleavage of pro-EaCDCL proteins.
(A) Crystal structure of pro-EaCDCL^L (PDB ID:6XD4) in cartoon representation, displaying the activation loop (red), preceding N-terminal region (navy), YGR motif (green) and diglycine motif (yellow spheres). Left inset, rotated -90° on y-axis and zoomed in on D2 region highlighting the hydrogen bonds (dashed black lines) between the preceding N-terminal region and the rest of EaCDCL^L. **(B)** Crystal structure of pro-EaCDCL^S (PDB ID: 8G32), colored as per (A). Left inset, rotated -90° on y-axis and zoomed in on D2 region highlighting the hydrogen bonds (dashed black lines) between the preceding N-terminal region and the rest of

EaCDCL^{S}. Right inset, rotated-90° on y-axis and zoomed in on D3 region highlighting the hydrogen bonds between the activation loop and the core D3 β-sheet. **(C)** SDS-PAGE analysis of purified pro-EaCDCL^{L} (left panel) and act-EaCDCL^{L} (right panel). **(D)** LC-MS analysis of the intact mass for purified pro-EaCDCL^{L} (beige) and act-EaCDCL^{L} (cyan) revealed loss of the full-length protein (55.85 kDa) and a major species of 50.15 kDa following activation. **(E)** SEC-MALS analysis of pro-EaCDCL^{L} (beige) and act-EaCDCL^{L} (cyan) using a Superdex 200 Increase 5/150 column in 20 mM HEPES pH 7.5, 150 mM NaCl, 1 mM TCEP, 0.1% sodium azide. Curves represent light scattering data, with spherical data points corresponding to the calculated molar mass across the span on the eluted peak. The estimated molar mass for pro-EaCDCL^{L} and act-EaCDCL^{L} was 53.4 ± 0.2 kDa and 52.4 ± 0.2 kDa, confirming both species to be monomeric in solution. **(F)** SDS-PAGE analysis of purified pro-EaCDCL^{S} (left panel) and act-EaCDCL^{S} (right panel). **(G)** LC-MS analysis of the intact mass for purified pro-EaCDCL^{S} (blue) and act-EaCDCL^{S} (red) revealed loss of the full-length protein (39.46 kDa) and a major species of 31.58 kDa following activation.

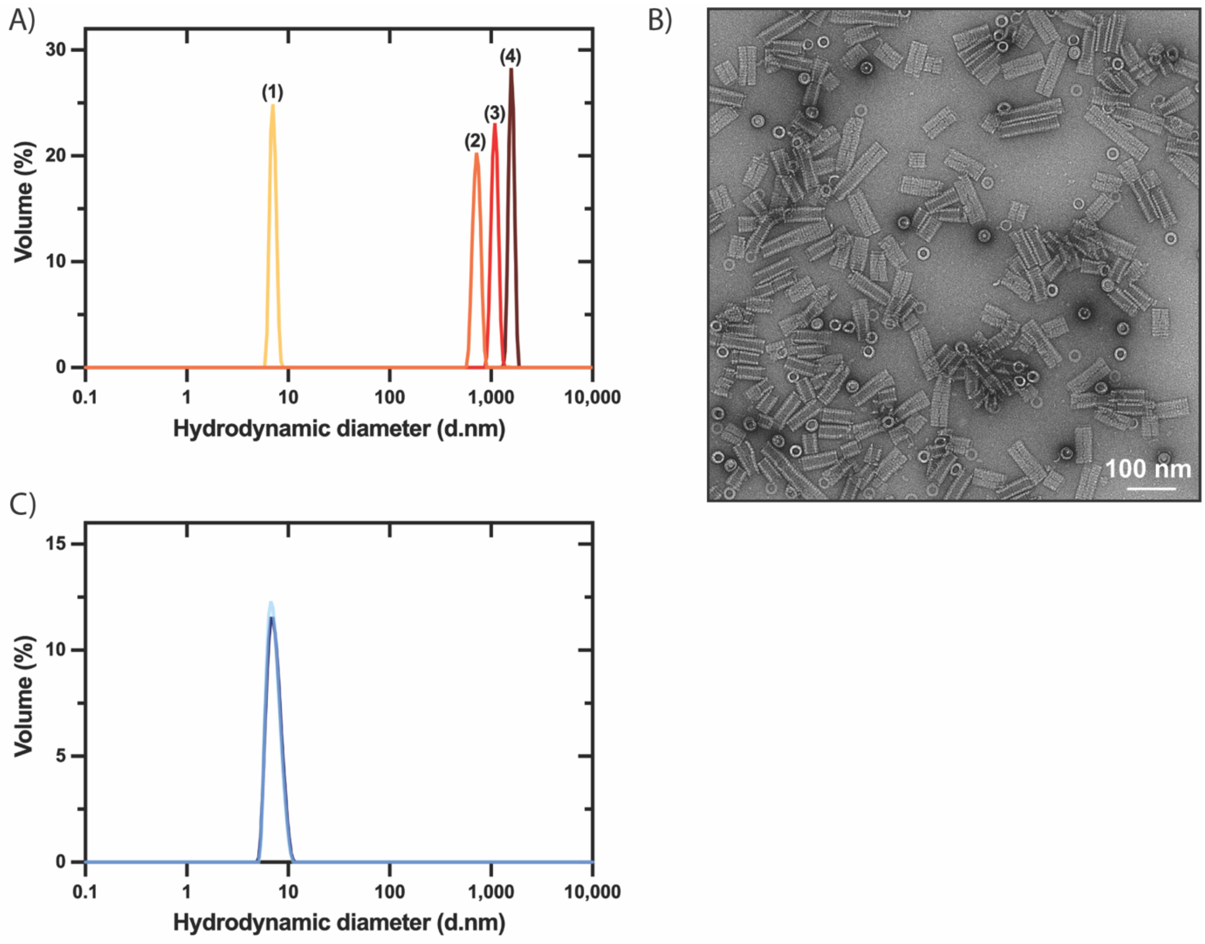

Fig. S2: Analysis of act-EaCDCL^{S} aggregation in solution.
(A) DLS of purified act-EaCDCL^{S} protein. Peak 1 (yellow) represents protein kept at 4°C, peak 2 (orange) represents protein kept at 24°C for 15 min, peak 3 (red) represents protein from peak 2 incubated at 24°C for a further 10 min, with peak 4 (burgundy) represents the protein sample depicted in peak 3 incubated at 37°C for 5 min. **(B)** Representative micrographs from negative-stain EM of act-EaCDCL^{S} (0.6 µM) protein incubated at 37°C for 20 min. **(C)** DLS of purified pro-EaCDCL^{S} protein incubated at 24°C for 15 min (light blue) or 16 hours (dark blue) does not reveal substantial signs of aggregation.

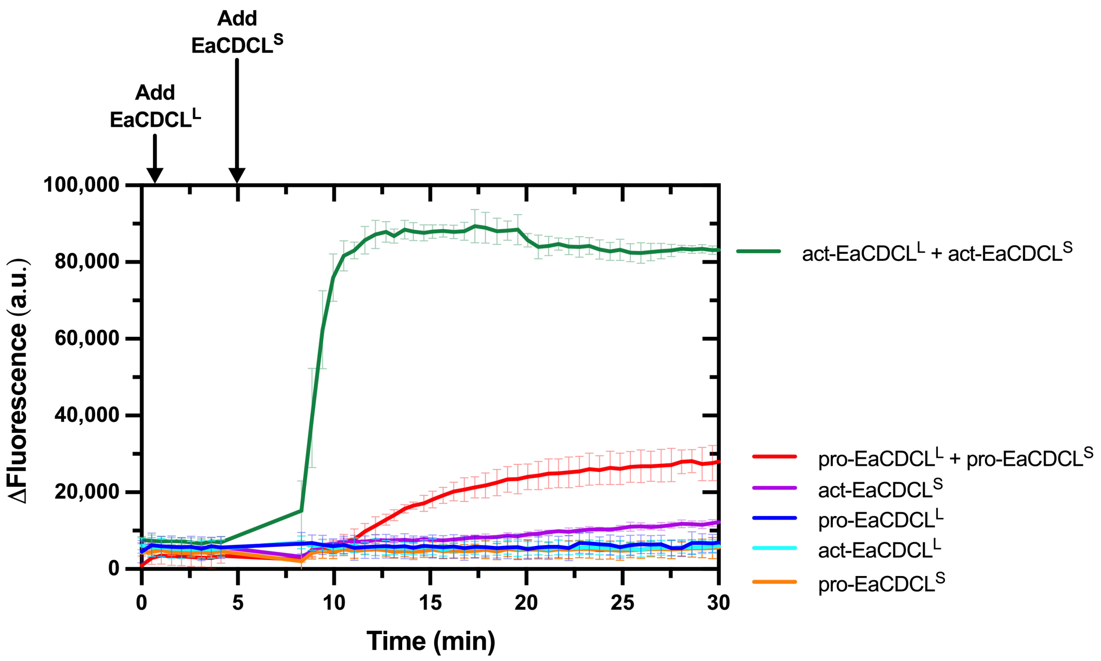

Fig. S3: Pore-forming activity of purified EaCDCL proteins used for structural analysis. Kinetic release of fluorescent marker (carboxyfluorescein, CF) from POPC liposomes (100 nm) when treated with either act-EaCDCL^L and act-EaCDCL^S (1 μM, 1:1 molar stoichiometry, green line), pro-EaCDCL^L and pro-EaCDCL^S (1 μM, 1:1 molar stoichiometry, red line), act-EaCDCL^L (1 μM, light blue line), pro-EaCDCL^L (1 μM, dark blue line), act-EaCDCL^S (1 μM, purple line) or pro-EaCDCL^S (1 μM, orange line). As pore-formation occurs, CF is released from the liposome, dequenching its emission and increasing the change in fluorescent signal. In all cases, EaCDCL^L protein (or buffer) was added to liposomes and allowed to incubate in the plate reader for 5 min at 37°C before the addition of EaCDCL^S (or buffer). No change in the emission is observed when the CF liposomes are incubated alone over the time frame of the experiment (not shown). Values plotted above are buffer corrected and represent the mean +/- SD of three replicates.

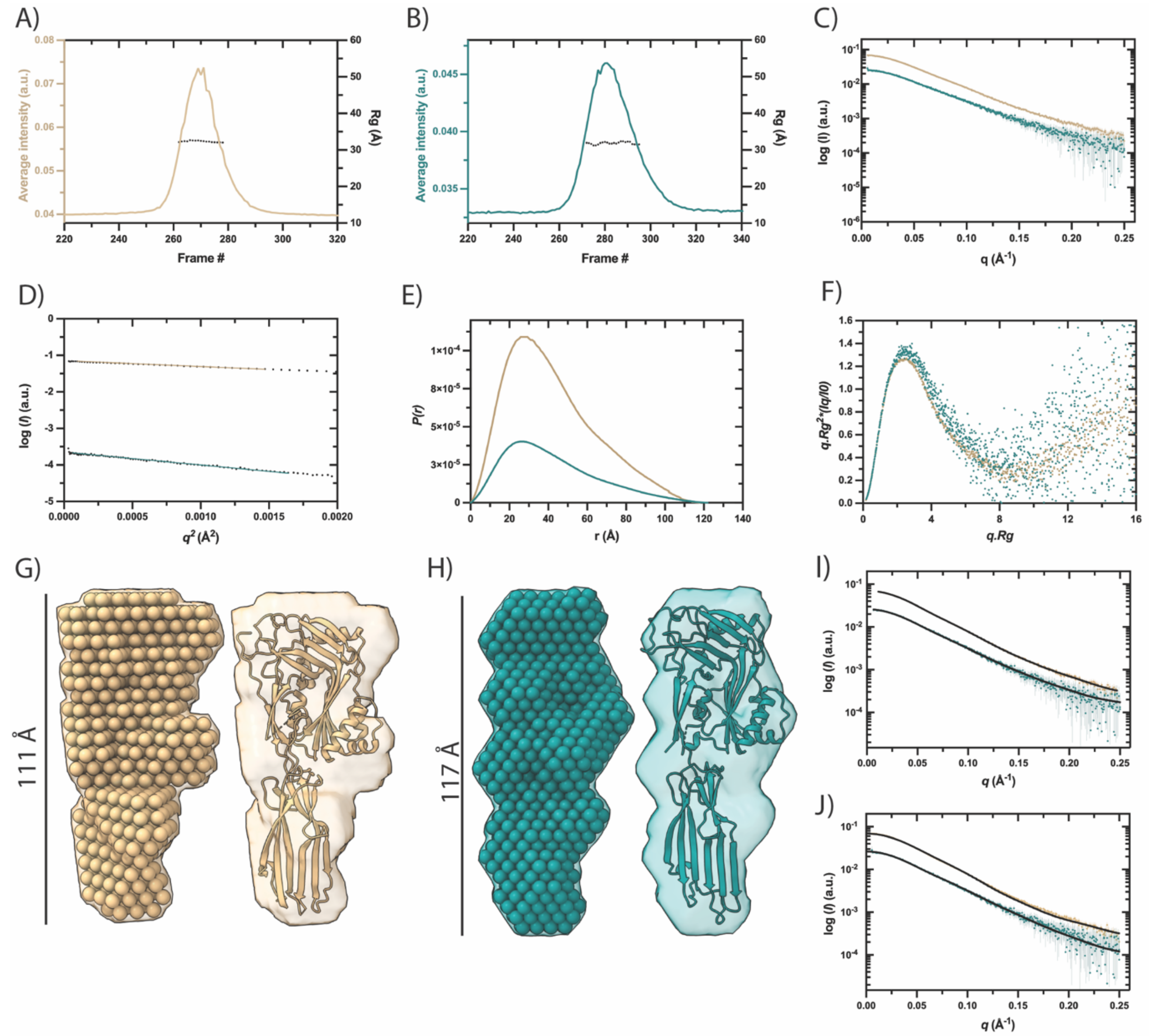

Fig. S4: SEC-SAXS analysis of EaCDCL^L.

(A) SEC-SAXS elution profile for pro-EaCDCL^L (10 mg/mL). **(B)** SEC-SAXS elution profile for act-EaCDCL^L (5 mg/mL). **(C)** Buffer-subtracted scattering profiles for pro-EaCDCL^L (beige) and act-EaCDCL^L (cyan). **(D)** Guinier plot for pro-EaCDCL^L (beige) and act-EaCDCL^L (cyan) revealing a linear fit and a R_g value of 32.40 ± 0.11 Å and 33.74 ± 0.27 Å, respectively. **(E)** *P(r)* plot for pro-EaCDCL^L (beige) (R_g of 33.57 ± 0.07 Å, D_{max} of 117.9 Å) and act-EaCDCL^L (cyan) (R_g of 33.76 ± 0.03 Å, D_{max} of 120.7 Å). **(E)** Dimensionless Kratky plot for scattering from pro-EaCDCL^L (beige) and act-EaCDCL^L (cyan). **(G)** *Ab initio* bead model obtained from pro-EaCDCL^L SEC-SAXS data (left) and superimposed with pro-EaCDCL^L crystal structure (right). **(H)** *Ab initio* bead model obtained from act-EaCDCL^L SEC-SAXS data (left) and superimposed with act-EaCDCL^L crystal structure (right). **(I)** Fit of *ab initio* bead models to experimental scattering data for pro-EaCDCL^L (χ^2 = 1.155) (beige) and act-EaCDCL^L (χ^2 = 1.247) (cyan). **(J)** Comparison of experimental scattering data for pro-EaCDCL^L (beige) and act-EaCDCL^L (cyan) with theoretical scattering curves, generated using CRYSOL, for pro-EaCDL^L crystal structure (χ^2 = 5.20) and act-EaCDCL^L crystal structure (χ^2 = 1.16).

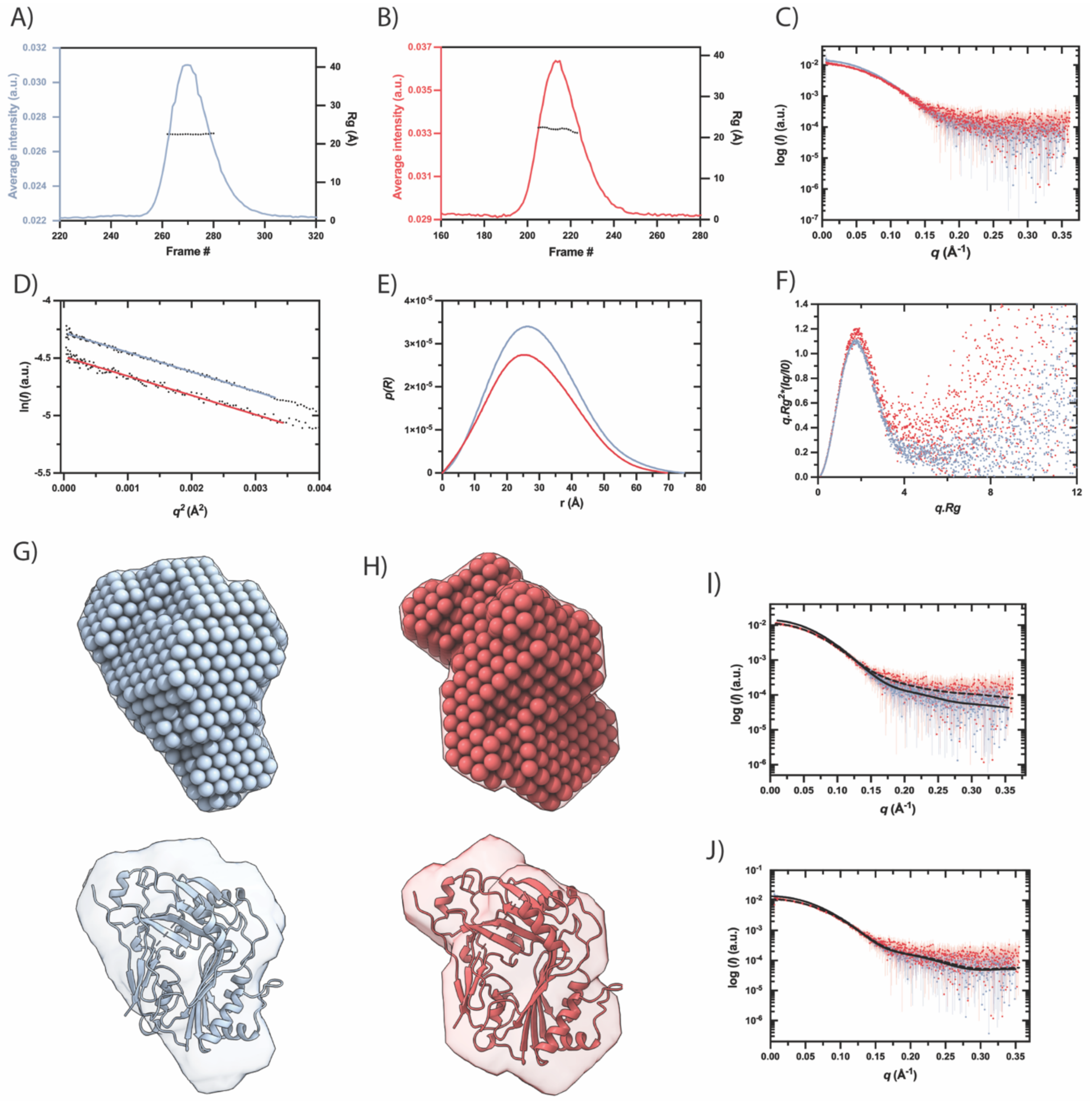

Fig. S5: SEC-SAXS analysis of EaCDCL^S.

(A) SEC-SAXS elution profile for pro-EaCDCL^S (5.0 mg/mL). **(B)** SEC-SAXS elution profile for act-EaCDCL^S (3.5 mg/mL). **(C)** Buffer-subtracted scattering profiles for pro-EaCDCL^S (light blue) and act-EaCDCL^S (red). **(D)** Guinier plot for pro-EaCDCL^S (light blue) and act-EaCDCL^L (red) revealing a linear fit and a R_g value of 22.52 ± 0.11 Å and 22.11 ± 0.27 Å, respectively. **(E)** *P(r)* plot for pro-EaCDCL^S (light blue) (R_g of 22.54 ± 0.10 Å, D_{max} of 74.8 Å) and act-EaCDCL^S (red) (R_g of 21.89 ± 0.16 Å, D_{max} of 69.6 Å). **(E)** Dimensionless Kratky plot for scattering from pro-EaCDCL^S (light blue) and act-EaCDCL^S (red). **(G)** *Ab initio* bead model obtained from pro-EaCDCL^S SEC-SAXS data (top) and superimposed with pro-EaCDCL^S crystal structure (bottom). **(H)** *Ab initio* bead model obtained from act-EaCDCL^S SEC-SAXS data (top) and superimposed with pro-EaCDCL^S crystal structure (bottom). **(I)** Fit of *ab initio* bead models to experimental scattering data for pro-EaCDCL^S (χ^2 = 1.103) (light blue, solid black line) and act-EaCDCL^S (χ^2 = 1.062) (red, dashed black line). **(J)** Comparison of theoretical scattering curve generated using CRYSOL for pro-EaCDCL^S crystal structure (black line) with experimental scattering data for pro-EaCDCL^S (light blue) (χ^2 = 1.20) and act-EaCDCL^S (red) (χ^2 = 1.32).

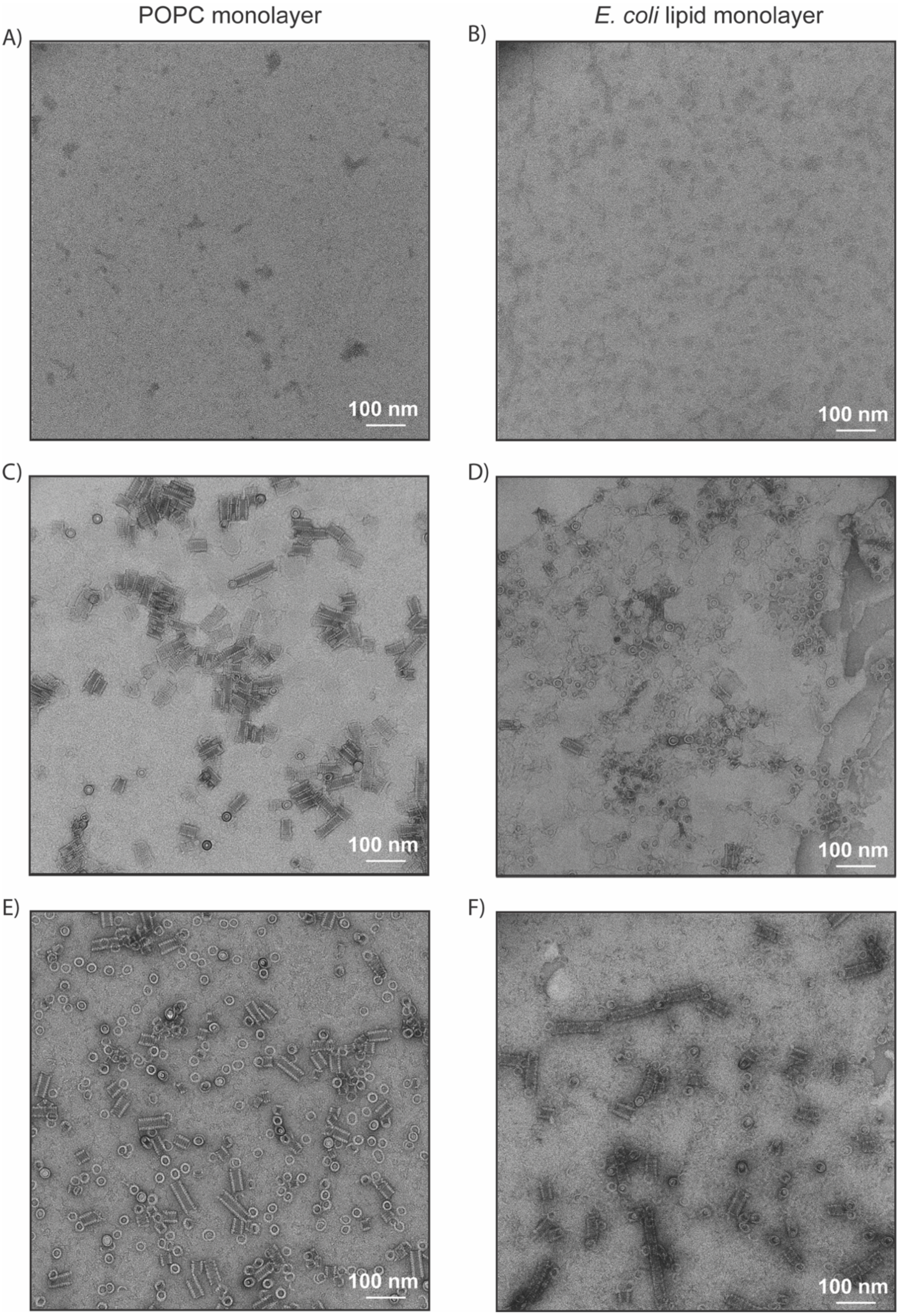

Fig. S6: Visualization of EaCDCLs on lipid monolayers by negative-stain TEM. Representative micrographs from negative-stain EM of act-EaCDCL^{L} (0.3 μM) protein on a (A) POPC lipid monolayer or (B) *E. coli* lipid extract monolayer, act-EaCDCL^{S} (0.3 μM) protein on a (C) POPC lipid monolayer or (D) *E. coli* lipid extract monolayer or act-EaCDCL^{L} (0.15 μM) and act-EaCDCL^{S} (0.3 μM) proteins together on a (E) POPC lipid monolayer or (F) *E. coli* lipid extract monolayer. For all samples, protein was incubated on the monolayer for 15-20 min before grids were stained and imaged.

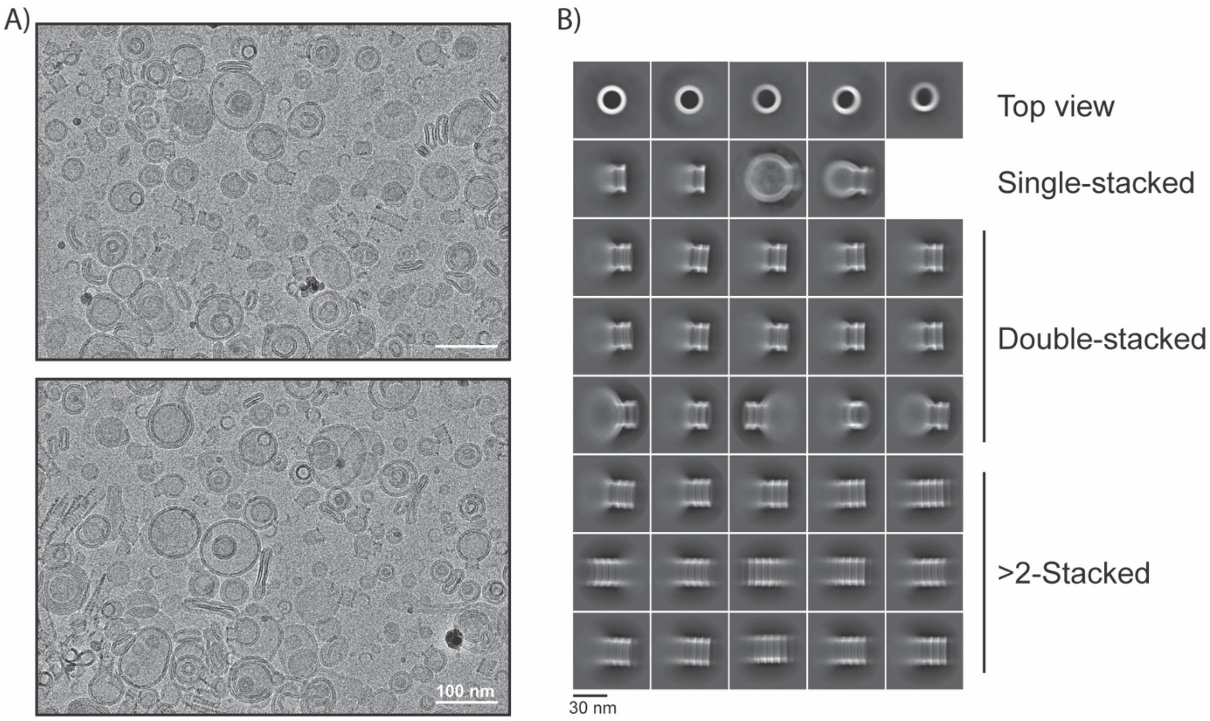

Fig. S7: Example micrographs and 2D class averages from cryo-EM analysis.
(A) Motion corrected micrographs of vitrified EaCDCL-proteoliposomes. Grids were prepared using a proteoliposome sample containing POPC liposomes (3.95 mM), act-EaCDCL^{L} (3.95 μM) and act-EaCDCL^{S} (7.9 μM), incubated at 37°C for 20 min. **(B)** Representative 2D class averages from cryo-EM dataset collected, revealing the presence of single-stacked pore complexes, double-stacked complexes and elongated stacked oligomers.

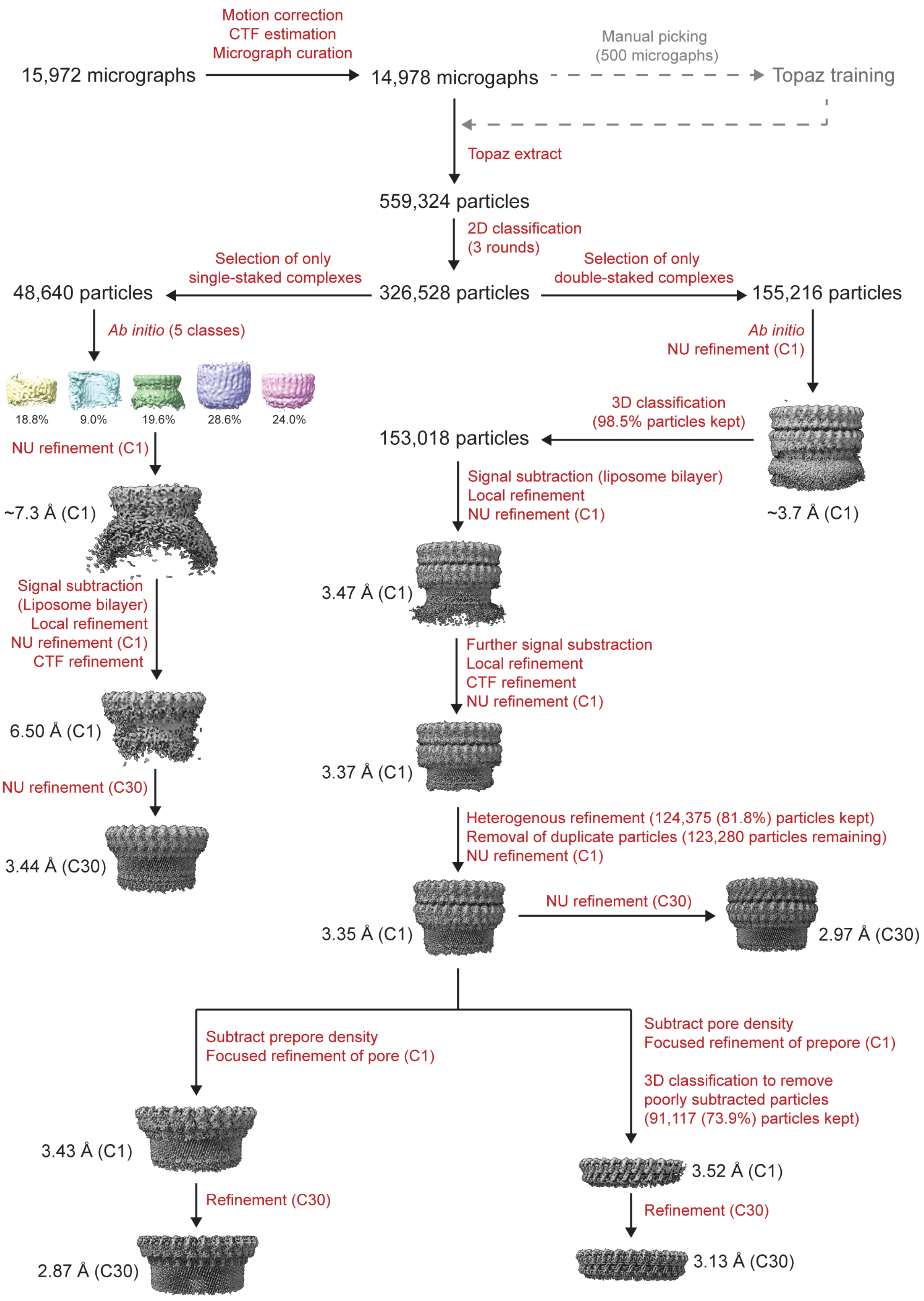

Fig. S8: Cryo-EM data processing workflow.
Workflow for the image processing of cryo-EM data of EaCDCLs on the surface of liposomes. Sharpened maps after the indicated processing steps are shown in grey.

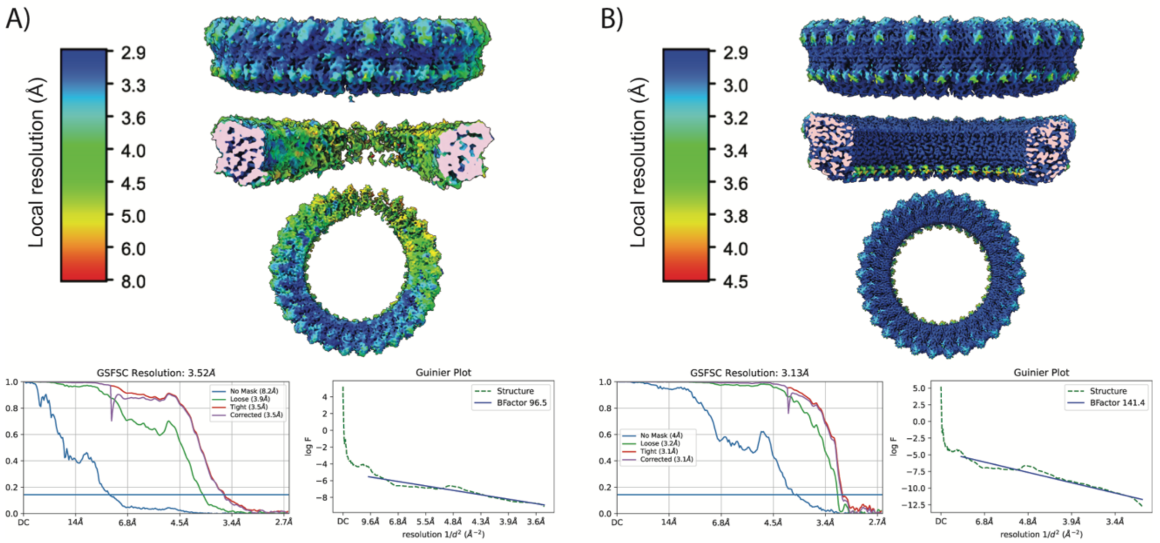

Fig. S9: Local resolution, FSC curves and Guinier plots for the cryo-EM maps of the EaCDCL prepore-like complex.

(A) 3D reconstruction (C1 symmetry) of the prepore-like complex, generated from double-stacked particles containing the inserted pore and prepore, colored according to local resolution (2.9 – 8.0 Å). Gold-standard Fourier shell correlation (FSC) curve (bottom left), calculated from two independent half-maps, reveals an overall resolution of 3.52 Å (FSC = 0.143). The large difference between the no mask and mask curves is likely due to remaining traces of the low-resolution signal from the liposome bilayer. Guinier plot (bottom right) indicates a sharpening B-factor of 96.5. **(B)** 3D reconstruction (C30 symmetry) of the prepore-like complex, generated from double-stacked particles containing the inserted pore and prepore, colored according to local resolution (2.9 – 4.5 Å). Gold-standard FSC curve (bottom left), calculated from two independent half-maps, reveals and overall resolution of 3.13 Å (FSC = 0.143). Guinier plot (bottom right) indicates a sharpening B-factor of 141.4.

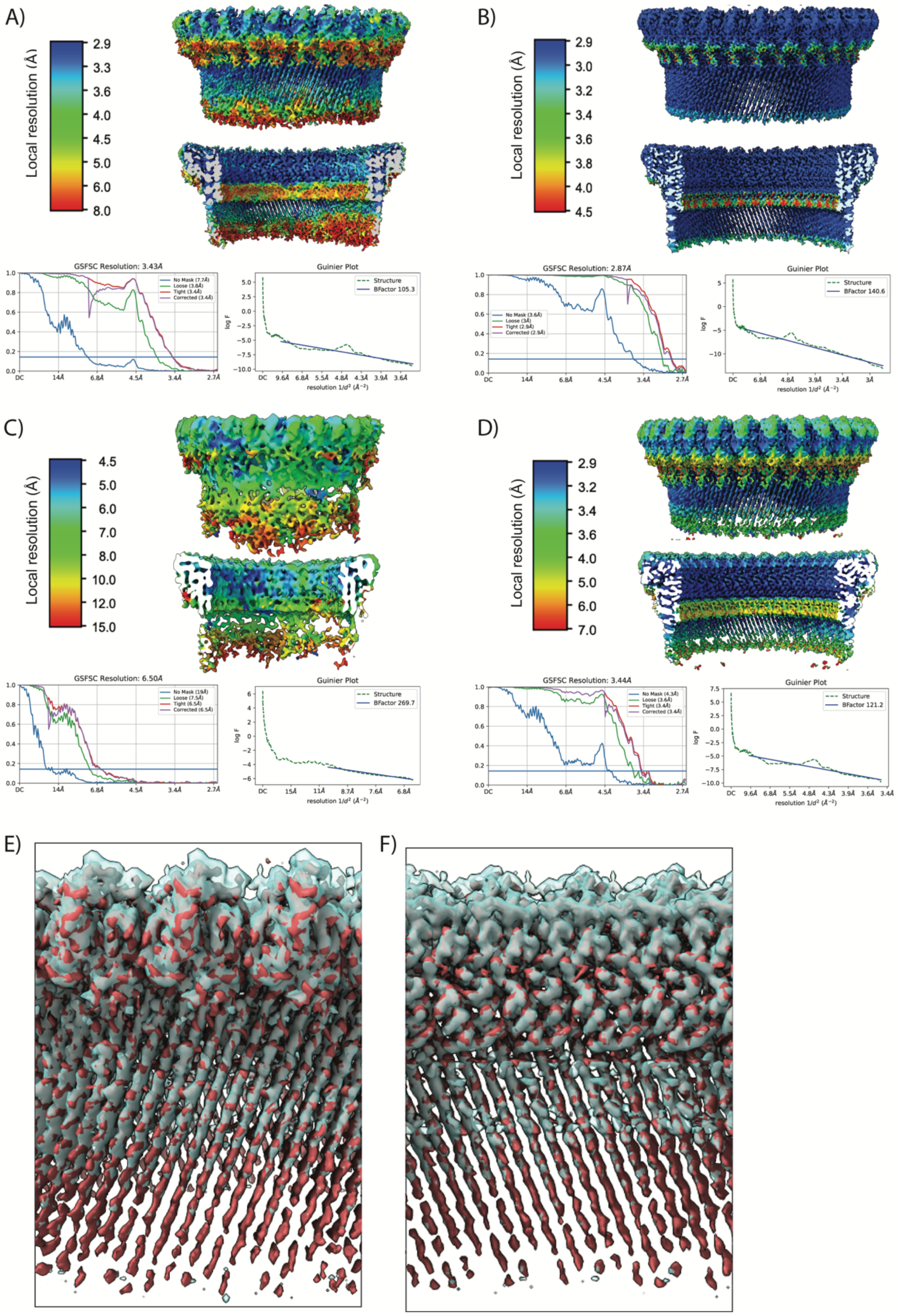

Fig. S10: Local resolution, FSC curves and Guinier plots for the cryo-EM maps of the EaCDCL pore complex.

(A) 3D reconstruction (C1 symmetry) of the inserted pore complex, generated from double-stacked particles containing the inserted pore and prepore, colored according to local resolution (2.9 – 8.0 Å). Gold-standard Fourier shell correlation (FSC) curve (bottom left), calculated from two independent half-maps, reveals an overall resolution of 3.43 Å (FSC = 0.143). The

large difference between the no mask and mask curves is likely due to remaining traces of the low-resolution signal from the liposome bilayer. Guinier plot (bottom right) indicates a sharpening B-factor of 105.3. **(B)** 3D reconstruction (C30 symmetry) of the inserted pore complex, generated from double-stacked particles containing the inserted pore and prepore, colored according to local resolution (2.9 – 4.5 Å). Gold-standard FSC curve (bottom left), calculated from two independent half-maps, reveals an overall resolution of 2.87 Å (FSC = 0.143). Guinier plot (bottom right) indicates a sharpening B-factor of 140.6. **(C)** 3D reconstruction (C1 symmetry) of the inserted pore complex, generated from non-stacked particles as a control for maps generated from double-stacked particles, colored according to local resolution (4.5 – 15.0 Å). Gold-standard FSC curve (bottom left), calculated from two independent half-maps, reveals an overall resolution of 6.50 Å (FSC = 0.143). Guinier plot (bottom right) indicates a sharpening B-factor of 269.7. **(D)** 3D reconstruction (C30 symmetry) of the inserted pore complex, generated from non-stacked particles, colored according to local resolution (2.9 – 7.0 Å). Gold-standard FSC curve (bottom left), calculated from two independent half-maps, reveals and overall resolution of 3.44 Å (FSC = 0.143). Guinier plot (bottom right) indicates a sharpening B-factor of 121.2. **(E-F)** Overlay of a section of the cryo-EM maps of the EaCDCL pore derived from the double-stacked particle stack (red) or the control, single-stacked particle stack (cyan). **(E)** View from the exterior of the pore. **(F)** View of the interior of the pore.

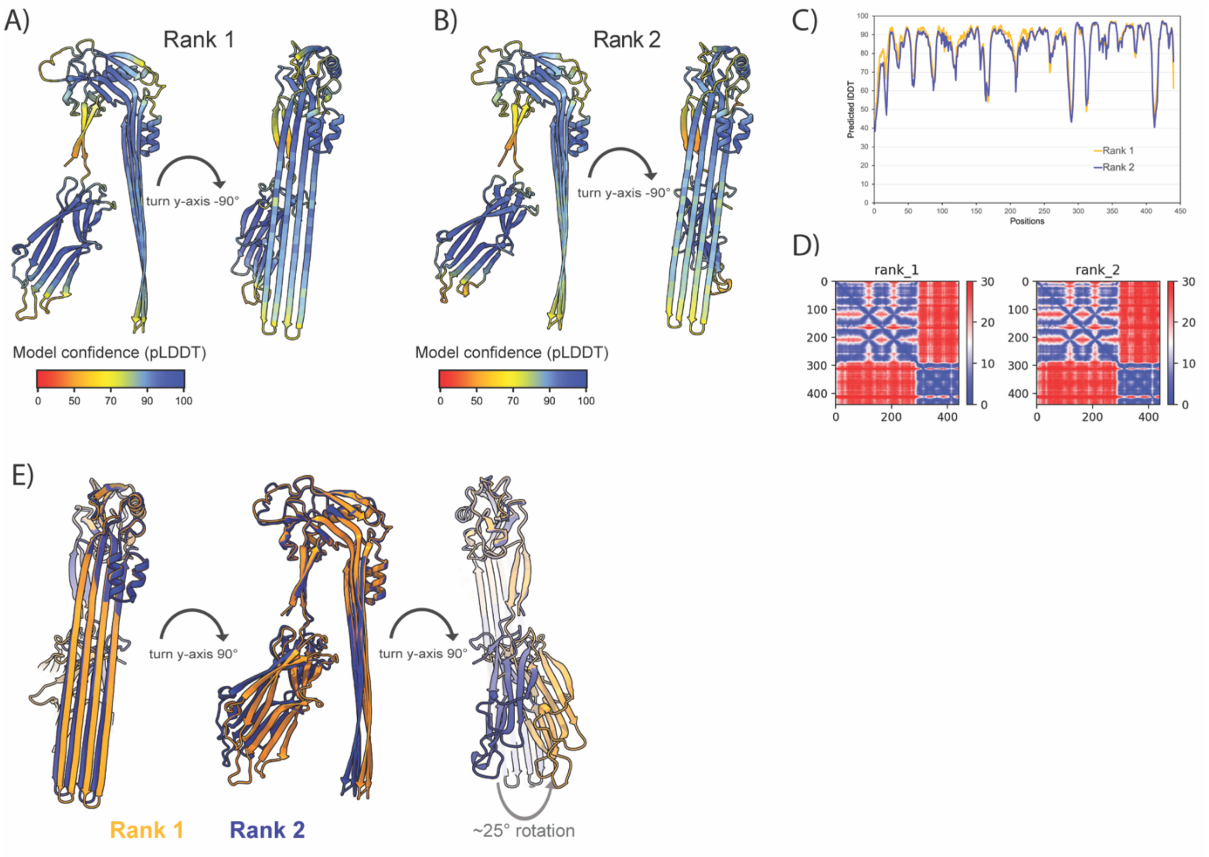

Fig. S11: AlphaFold2 modeling of EaCDCL^{L} in the pore state.
(A) The highest ranking predicted model (rank 1) generated by AlphaFold2 using EaCDCL^{S} and PLY in the pore form, and D4 of the pro-EaCDCL^{L} structure as templates. The model is shown in cartoon representation colored by predicted confidence (pLDDT) as per the color key shown. **(B)** The second highest ranking predicted model (rank 2) generated by AlphaFold2, colored as per (A), **(C)** Plot of predicted confidence (pLDDT) per position for rank 1 (orange) and rank 2 (blue) models. **(D)** Predicted alignment error (PAE) plots of the models generated, with scored residue on x-axis and aligned residue on y-axis. **(E)** Structural alignment of the rank 1 (orange) and rank 2 (blue) predicted models, showing differing positions of D4 with respect to D1, D2 and D3 domains.

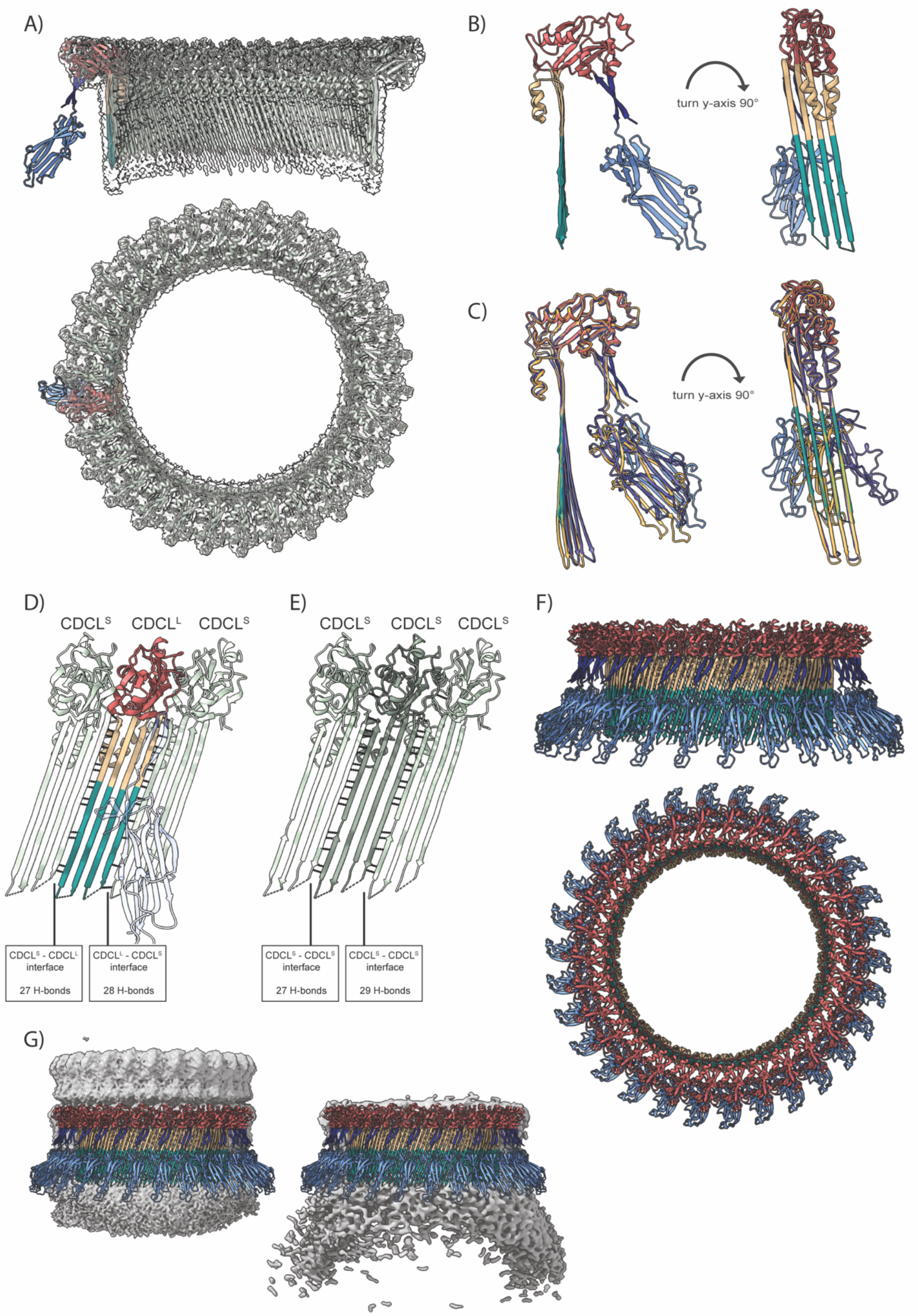

Fig. S12: Refinement and analysis of EaCDCL^L in the pore state.
(A) The top ranking AlphaFold2 model (rank 1) of EaCDCL^L in the pore state was subject to modelling and refinement into the 30-mer cryo-EM map, in place of a single CDCL^S subunit. The 30-mer pore is shown overlaid with the cryo-EM map (transparent grey) with EaCDCL^S subunits in pale green and the single EaCDCL^L subunit colored according to domain: D1 (pink), D2 (navy), D3 (beige and cyan), D4 (light blue) and αHBs/TMH regions (cyan). Top,

clipped view of the pore from the side. Bottom, top view of the pore. **(B)** Cartoon representation of the refined EaCDCL^L pore model, colored as per (A). **(C)** Overlay of the refined EaCDCL^L pore model, colored as per (A), with the AlphaFold2 models described in Fig. S11 (colored in orange and blue). **(D)** Putative hydrogen bonds between EaCDCL^L and neighboring subunits in the transmembrane pore. **(E)** Hydrogen bonds between EaCDCL^S subunits in the transmembrane pore. **(F)** Cartoon representation of the modelled transmembrane pore composed of 30 EaCDCL^L subunits. Top, side view. Bottom, top view of the pore, rotated 90° around y-axis from side view. **(G)** Overlay of the 30-mer EaCDCL^L pore model with initial C1 cryo-EM reconstructions of the double-stacked pore (left) or single-stacked pore (right).

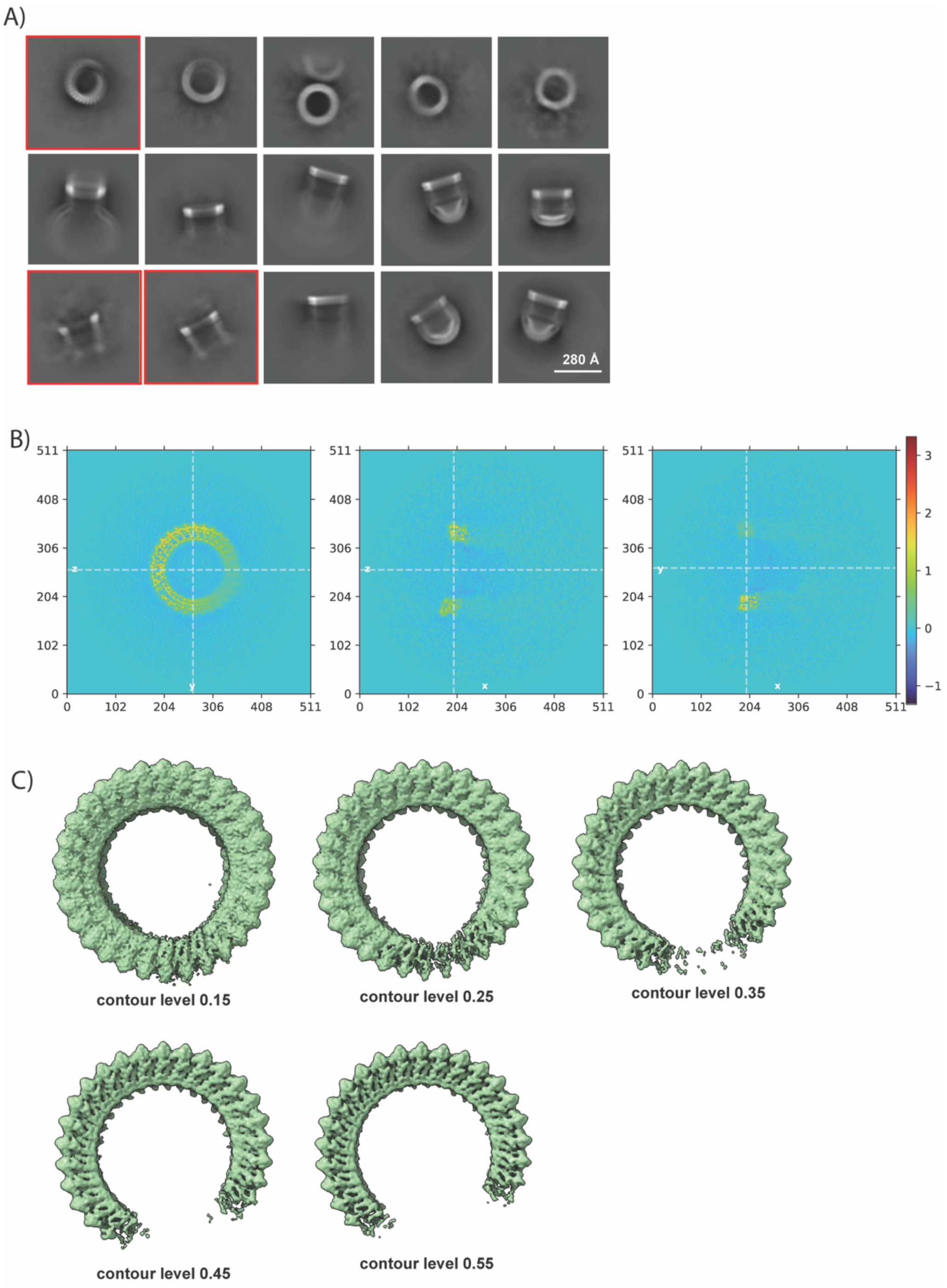

Fig. S13: The prepore-like oligomer, derived from double-stacked pores, is an incomplete ring.

(A) 2D class averages of the particle stack for the prepore-like oligomer, derived from the primary double-stacked particle stack (liposome-signal subtracted) and after rounds of signal subtraction for the inserted pore. Averages that represent putative non-circular and/or complete oligomers are highlighted by red boxes. (B) Real-space slices for the 3D density of the prepore-like oligomer reconstruction using the particle stack in (A) and no symmetry applied (C1 symmetry). (C) The raw, unsharpened prepore-like oligomer reconstruction (C1 symmetry) displayed at varying contour levels.

Table S1: Data collection and refinement statistics for the act-CDCL^L crystal structure.

	**Act-EaCDCL^L**
**Data collection**	
Wavelength (Å)	0.9537
Unit cell parameters:	
a, b, c (Å)	61.7, 24.5, 300.2
α, β, γ (°)	90.0. 93.2, 90.0
Space group	*I*2
Resolution range (Å)	44.22 - 2.49 (2.59 - 2.49)
Unique reflections	16,351 (1703)
Completeness (%)	99.2 (94.5)
Multiplicity	4.6 (4.7)
Mean I /σ I	7.1 (1.8)
$CC_{1/2}$	0.989 (0.557)
R_{pim}	0.088 (0.496)
Wilson B-factor	36.31
**Refinement**	
Reflections used in refinement	16351 (1485)
Reflections used for R_{free}	816 (66)
R_{work}	0.2075 (0.2775)
R_{free}	0.2479 (0.3583)
No. non-hydrogen atoms	3537
macromolecules	3492
ligands	0
solvent	45
Protein residues	436
R.m.s deviations	
Bond lengths (Å)	0.002
Bond angles (°)	0.47
Ramachandran plot	
Favored (%)	96.30
Allowed (%)	3.70
Outliers (%)	0.00
Rotamer outliers (%)	5.36
Clashscore	2.31
Average B-factor (Å^2)	43.89
macromolecules	44.05
solvent	31.39
PDB ID	8G33

Statistics for the highest-resolution shell are shown in parentheses.

Table S2: SEC-SAXS data collection and analysis parameters.

	**Pro-EaCDCL^{L}**	**Act-EaCDCL^{L}**	**Pro-EaCDCL^{S}**	**Act-EaCDCL^{S}**
**SAXS data-collection parameters**				
Instrument/source	Australian Synchrotron SAXS/WAXS beamline equipped with Pilatus 2M detector and sheathflow cell for SEC-SAXS			
Wavelength (Å)	1.078	1.033	1.078	1.033
Beam energy (kEv)	11.50	12.0	11.50	12.0
Beam size (μm)	250 × 130			
Sample-to-detector distance (mm)	2590	2490	2590	2490
q-measurement range (Å^{-1})	0.006 - 0.54	0.006 - 0.59	0.005 - 0.62	0.006 - 0.59
Absolute scaling method	Comparison with scattering from 1 mm pure water			
Normalization	To transmitted intensity from beamstop counter			
Exposure time	1 s measurements from SEC-SAXS elution			
Sample temperature (K)	295	295	295	283
**Sample details and SEC-SAXS parameters**				
Column	Superose 6 5/150 Increase	Superdex 200 5/150 Increase	Superdex 75 5/150 Increase	
Flow rate (mL/min)	0.35	0.40	0.40	0.40
Injection volume (μL)	50			
Concentration (mg/mL)	10.0	5.0	5.0	3.5
Solvent	20 mM HEPES pH 7.5, 150 mM NaCl, 1 mM TCEP, 0.1% NaN_3		20 mM HEPES pH 7.5, 150 mM NaCl, 2 mM $CaCl_2$, 0.1% NaN_3	
**Software employed**				
SAXS data reduction	$I(q)$ vs q using Scatterbrain 3.0.2, SEC-SAXS solvent subtraction using *CHROMIXS* from *ATSAS* 3.0.4			
Basic analysis (Guinier, *P(r)*, molecular mass)	*PRIMUS* from *ATSAS 3.0.4*, *GNOM* from *ATSAS 3.0.4*			
Ab initio modelling	*DAMMIF* from *ATSAS* 3.0.4, *DAMAVER* from *ATSAS* 3.0.4, *DAMMIN* from *ATSAS* 3.0.4			
Calculation of theoretical intensities	*CRYSOL* from *ATSAS* 3.0.4			
Three-dimensional graphic model representations	ChimeraX-1.5			
**Structural parameters**				
**Guinier analysis**				
Rg (Å)	32.40 ± 0.11	31.99 ± 0.27	22.52 ± 0.11	22.11 ± 0.27
I(0) (cm^{-1})	0.070 ± 1 x 10^{-4}	0.026 ± 1 x 10^{-5}	0.014 ± 4 x 10^{-5}	0.011 ± 8 x 10^{-5}
qRg min,max	0.35, 1.24	0.21, 1.30	0.25, 1.29	0.18, 1.30
***P(r)* analysis**				
Rg (Å)	33.57 ± 0.11	33.78 ± 0.30	22.54 ± 0.10	21.89 ± 0.16
I(0) (cm^{-1})	0.0703 ± 0.15 x 10^{-3}	0.0261 ± 0.14 x 10^{-3}	0.0138 ± 0.41 x 10^{-4}	0.0111 ± 0.68 x 10^{-4}
Dmax (Å)	117.9	121.5	74.8	69.6
Porod volume (Å^3)	72140	65036	57859	48965

**Shape modelling**				
***DAMMIF* (10 calculations, default parameters)**				
q range for fitting (Å)	0.006 – 0.247	0.006 – 0.250	0.005 – 0.355	0.006 – 0.361
Symmetry, anisotropy assumptions	P1, none	P1, none	P1, none	P1, none
Constant adjustment to intensities	0.702 x 10^{-4}	0.551 x 10^{-4}	0.340 x 10^{-4}	0.782 x 10^{-4}
NSD (standard deviations)	0.879 (0.062)	1.017 (0.053)	0.701 (0.047)	0.812 (0.078)
χ^2 range	1.155 - 1.160	1.256 - 1.260	1.100 -1.108	1.056 - 1.060
Resolution from *SASRES* (Å)	38	33	20	25
***DAMMIN* (default parameters, DAMSTART average as search volume**				
q range for fitting (Å)	0.006 – 0.247	0.006 – 0.250	0.005 – 0.355	0.006 – 0.361
Symmetry, anisotropy assumptions	P1, none	P1, none	P1, none	P1, none
χ^2	1.155	1.247	1.103	1.062
Constant adjustment to intensities	0.523 x 10^{-4}	0.556 x 10^{-5}	3.410 x 10^{-5}	6.449 x 10^{-5}
**Atomic modelling**				
***CRYSOL* (no constant subtraction)**				
Structure	Pro-EaCDCL^{L} crystal structure (PDB ID: 6XD4)	Act-EaCDCL^{L} crystal structure	Pro-EaCDCL^{S} crystal structure (chain A only) (PDB ID: 8G32)	
χ^2	5.20	1.16	1.20	1.32
Calculated *R*g (Å)	33.10	34.33	22.09	21.22

Table S3: Cryo-electron microscopy data collection, refinement and validation statistics.

	**Double-stacked complex** (C1)	**Double-stacked complex** (C30)	**Inserted pore complex** (C1, from double-stacked particles)	**Inserted pore complex** (C30, from double-stacked particles)	**Inserted pore complex** (C1, from single-stacked particles)	**Inserted pore complex** (C30, from single-stacked particles)	**Prepore-like complex** (C1, from double-stacked particles)	**Prepore-like complex** (C30, from double-stacked particles)
**Data collection and image processing**								
Magnification	64,000							
Electron energy (kV)	300							
Energy filter slit width (eV)	20							
Total exposure dose ($e^-/Å^2$)	50							
Defocus range (µm)	0.8 - 2.0							
Pixel size (Å)	1.32							
Total number of micrographs	15,971							
Symmetry imposed	C1	C30	C1	C30	C1	C30	C1	C30
Initial number of particles	326,528							
Final number of particles	123,280	123,280	122,300	122,300	9,532	9,532	91,117	91,117
Map resolution (Å)	3.35	2.97	3.43	2.87	6.50	3.44	3.52	3.13
FSC threshold	0.143	0.143	0.143	0.143	0.143	0.143	0.143	0.143
EMDB ID	EMD-45448	EMD-45449	EMD-45450	EMD-45451	EMD-45454	EMD-45455	EMD-45452	EMD-45453
**Model building and refinement**								
Initial model used				Pro-EaCDCL^S monomer (PDB ID: 8G32, chain A)				Pro-EaCDCL^S monomer (PDB ID: 8G32, chain A)
Map sharpening B factor ($Å^2$)				-140.6				-141.4
No. non-hydrogen atoms				58,080				62,430
Protein residues				7,590				8,160
Ligands				CA: 30				CA: 30
Model-to-map fit (CC)				0.80				0.84
Q-score (overall)				0.5710				0.5450
R.m.s deviations:								
Bond lengths (Å)				0.004				0.003

Bond angles (°)		0.637		0.455
Molprobity score		1.53		1.45
Clashscore		8.18		7.10
Rotamer outliers (%)		0.93		0.00
Ramachandr-an plot				
Favored (%)		97.57		97.76
Allowed (%)		2.43		2.24
Outliers (%)		0.0		0.0
PDB ID		9CCP		9CCQ

Table S4: Structural comparison of EaCDCL^S in the pore state with other CDC, MACPF and gasdermin pore structures.

Protein name	PDB ID	Family	Organism	DALI Z-score	TM-score (normalized to EaCDCL^S)	Sequence identity (%)
PLY	5LY6	CDC	*Streptococcus pneumoniae*	15.2	0.841	22
C9	6DLW	MACPF	*Homo sapiens*	10.3	0.576	7
Mpf2Ba1	8B6W	MACPF	*Pseudomonas monteilii*	9.4	0.606	5
Perforin-2 (MPEG1)	8A1D	MACPF	*Mus musculus*	9.4	0.572	8
Perforin	7PAG	MACPF	*Mus musculus*	6.9	0.548	10
Bacterial gasdermin (bGSDM)	8SL0	Gasdermin	*Vitiosangium sp.*	8.7	0.478	9
Gasdermin-like protein rcd-1-1	8JYZ	Gasdermin	*Neurospora crassa*	9.7	0.412	6
Gasdermin-like protein rcd-1-2	8JYZ	Gasdermin	*Neurospora crassa*	9.7	0.397	8
*Tricho*GSDM	8JYW	Gasdermin	*Trichoplax adhaerens*	8.5	0.512	7
Gasdermin A3 (GSDMA3)	6CB8	Gasdermin	*Mus musculus*	8.0	0.437	11
Gasdermin D (GSDMD)	6VFE	Gasdermin	*Homo sapiens*	6.6	0.499	10
Gasdermin B (GSDMB)	8ET2	Gasdermin	*Homo sapiens*	4.0	0.434	4

Movie S1: Conformational changes during activation of EaCDCL^{L}.

Comparison of the pro-EaCDCL^{L} (PDB ID: 6XD4) and act-EaCDCL^{L} structures reveals movement of the D4 domain, relative to the D1, D2 and D3 domains, loss of the activation loop and preceding N-terminus and shortening of the β5 strand in the core D3 β-sheet.

Movie S2: Conformational change of EaCDCL^{S} during pore formation.

Loss of the activation loop observed in the pro-EaCDCL^{S} structure (PDB ID: 8G32) allows for formation of the prepore-like oligomer that displays straightening of the core D3 β-sheet and the β-to-α-transition of the β5 strand to form the HTH. Pore insertion involves the straightening of the α-HBs to form TMHs that create the ~167 Å β-barrel pore.
